# Vinegar intake in patients undergoing immune checkpoint inhibitor therapy: food frequency questionnaire study

**DOI:** 10.3389/fimmu.2025.1640603

**Published:** 2025-12-11

**Authors:** Hirotsugu Ariizumi, Miyuki Shimazui, Chisato Ogawa, Mika Kaneki, Nanaho Saito, Emiko Mura, Risako Suzuki, Toshiaki Tsurui, Nana Iriguchi, Tomoyuki Ishiguro, Yuya Hirasawa, Masahiro Shimokawa, Ryotaro Ohkuma, Yutaro Kubota, Atsushi Horiike, Satoshi Wada, Kiyoshi Yoshimura, Kouzou Murakami, Takuya Tsunoda

**Affiliations:** 1 Division of Medical Oncology, Department of Medicine, School of Medicine, Showa Medical University, Tokyo, Japan; 2 Division of Nutrition, Showa Medical University Hospital, Tokyo, Japan; 3 Department of Clinical Diagnostic Oncology, Clinical Research Institute for Clinical Pharmacology and Therapeutics, Showa Medical University, Tokyo, Japan; 4 Department of Clinical Immuno Oncology, Clinical Research Institute for Clinical Pharmacology and Therapeutics, Showa Medical University, Tokyo, Japan; 5 Division of Radiology, Department of Radiology, School of Medicine, Showa Medical University, Tokyo, Japan

**Keywords:** vinegar, acetic acid, immune checkpoint inhibitor, nutrition, food frequency questionnaire, dietary fiber

## Abstract

**Introduction:**

The gut microbiome is increasingly recognized as a key modulator of immune checkpoint inhibitor (ICI) efficacy. Dietary factors, particularly fibers, may influence the microbiome and thus affect the ICI response. Although Western studies have suggested a link between high fiber intake and better outcomes, this relationship remains unclear in Japanese populations with different dietary habits. This study investigated dietary components associated with ICI response in Japanese patients with cancer.

**Methods:**

In total, 32 patients with carcinomas treated with ICIs were enrolled. Nutritional customs before ICI infusion were analyzed using a food frequency questionnaire.

**Results:**

Among the 331 dietary items, only vinegar (acetic acid) intake showed an independent association with the treatment response. Higher vinegar consumption correlated with significantly lower odds of nonresponse (P = 0.017). In contrast, total and fermentable dietary fiber intake showed no significant association with ICI efficacy or survival outcomes.

**Conclusions:**

Higher vinegar intake is associated with better ICI response in Japanese patients, whereas fiber has a limited effect. Thus, tailored dietary strategies are needed for optimal outcomes.

## 1 Introduction

The efficacy of immune checkpoint inhibitors (ICIs) has been linked to the gut microbiota. Landmark studies in mice and humans have demonstrated that certain commensal bacteria could enhance antitumor immunity and improve ICI response (1, 2). Conversely, microbiome disruption (e.g., by antibiotics) before or during ICI therapy is associated with significantly worse survival outcomes in studies from our institute and others (3, 4). Previously, we had demonstrated that the overall survival of ICI-treated patients with antibiotics decreased by 70% compared with that of patients without antibiotics (4). Furthermore, *Turicibacter* and *Acidaminococcus* are the key microbiomes that help determine the clinical efficacy of ICI therapy in study from our institute (5). A recent clinical study in the USA reported that a high dietary fiber intake was correlated with prolonged survival in patients with melanoma receiving ICIs (6). High-fiber diets will promote a diverse (7), beneficial gut microbiome that can augment anticancer immune response (8), despite some opposing views (9, 10).

However, the gut microbiota composition varies greatly across different populations. Notably, the intestinal flora of Japanese individuals—characterized by a high abundance of *Bifidobacteria*—differs substantially from that of Western populations (11). Dietary habits and nutritional guidelines also differ by region (12), which may influence the microbiota and, in turn, ICI outcomes (13). To date, no study has specifically examined how contemporary Japanese diet patterns might affect the efficacy of ICI therapy. Given these gaps, the present study aimed to investigate whether pretreatment dietary habits in a Japanese patient cohort are associated with the clinical outcomes of ICI therapy. In particular, this study focused on dietary fiber intake—previously identified as beneficial in Western studies—and other diet components prevalent in Japan to identify any “favorite foods” that correlate with better ICI response in the context of Japanese food customs.

## 2 Material and methods

### 2.1 Study design and patients

A single-center prospective cohort study was conducted as an exploratory pilot study at Showa University Hospital (Tokyo, Japan) between December 2021 and June 2024. Patients with advanced cancers who were scheduled to receive their first ICI therapy were enrolled. Those who had prior cytotoxic chemotherapy were considered. A total of 50 patients provided consent, of whom 32 (men, n = 24; women, n = 8) completed the dietary survey and were included in the analysis. The median age was 71.5 (range, 59–86) years. Cancer types included esophageal carcinoma (n = 13), gastric carcinoma (n = 9), lung carcinoma (n = 4), duodenal carcinoma (n = 1), malignant mesothelioma (n = 1), uterine corpus carcinoma (n = 3), and Merkel cell carcinoma (n = 1) (**Table 1**). According to the standard regimens covered by public medical insurance, all patients received an ICI, either as monotherapy or in combination with other agents. Treatments included nivolumab (n = 16, including 1 patient with nivolumab + ipilimumab combination), pembrolizumab (n = 15), and avelumab (n = 1). Of the 32 patients, 24 received ICI therapy in combination with cytotoxic or targeted agents.

**Table 1:** Characteristics of the patients (n = 32).

Sex	
Male	24
Female	8
Age median (range)	71.5 (59–86)
Original cancer	
Esophageal carcinoma	13
Lung carcinoma	4
Gastric carcinoma	9
Duodenum carcinoma	1
Malignant mesothelioma	1
Corpus of the uterus	3
Merkel cell carcinoma	1
Regimen of ICI therapy	
Nivolumab-containing regimens	16
Nivolumab monotherapy	1
Nivolumab + SOX	7
Nivolumab + FOLFOX	4
Nivolumab + ipilimumab	4
Pembrolizumab-containing regimens	15
Pembrolizumab monotherapy	2
Pembrolizumab + 5FU + cisplatin	9
Pembrolizumab + Lenvatinib	3
Pembrolizumab + pemetrexed + carboplatin	1
Avelumab monotherapy	1
Max response of ICI therapy	
CR	3
PR	8
SD	13
PD	8

ICI, immune checkpoint inhibitor; SOX regimen consists of S1 and oxaliplatin; FOLFOX regimen consists of 5FU, levofolinate, and oxaliplatin.CR, complete response; PR, partial response; SD, stable disease; PD, progressive disease.

This study was conducted in accordance with the guidelines of the Declaration of Helsinki and was approved by the Ethics Committee of Showa University (Approval no. 21-064-A). Informed consent was obtained from all patients involved in the study.

### 2.2 Dietary assessment

Each patient’s habitual diet before ICI initiation was assessed using a validated, self-administered food frequency questionnaire (FFQ) developed for the Japan Public Health Center-based Prospective Study (14–16). This FFQ (185 questions long-form version, sold by Educational Software Co. Ltd., Hachiouji, Japan) was administered by nationally registered dietitians, and it captured the intake frequency and portion size of various foods in the previous year. Nutrient intakes were calculated using the 7th edition of the Standard Tables of Food Composition in Japan 2015. In total, 331 dietary variables were obtained for each patient, including total food weight, energy and nutrient intake, and specific food group amounts.

Notably, the FFQ’s default fiber calculations are based on the Prosky-modified method (AOAC991.42, AOAC 993.19, and AOAC985.29), which measures “dietary fiber” as mostly nondigestible polysaccharides but does not account for some fermentable components such as resistant starch, low-molecular-weight soluble fibers, and nondigestible oligosaccharides (17). To estimate the intake of the major fermentable fibers that were undercounted using the 2015 method, a *post hoc* calculation was performed. Specifically, the total dietary fiber from staple grain foods (rice, bread, noodles, and potatoes) was summed using updated values from the 8^th^ edition Standard Tables of Food Composition in Japan 2020, which adopted AOAC 2011.25 method (17). This allowed the inclusion of resistant starch and other fermentable dietary fiber fractions (e.g., arabinoxylan and β-glucan) predominantly present in grains (18–21). The English names of the Japanese food items were according to the English version of the 2015 Standard Tables of Food Composition in Japan.

### 2.3 Treatment response evaluation

The tumor response was assessed using the Response Evaluation Criteria in Solid Tumors (RECIST) version 1.1. The best objective response for each patient was categorized as complete response (CR), partial response (PR), stable disease (SD), or progressive disease (PD). For statistical analysis, the objective response rate (ORR) was defined as the proportion of patients achieving CR or PR versus nonresponse as SD or PD. The disease control rate (DCR) was defined as the proportion of patients achieving CR/PR/SD (disease control) versus PD. Immune-related adverse events were graded according to the Common Terminology Criteria for Adverse Events version 5.0.

### 2.4 Survival outcomes

Overall survival (OS) was defined as the time from the first ICI administration to death from any cause (or last follow-up). Progression-free survival (PFS) was defined as the time from ICI therapy initiation to radiographic or clinical disease progression, death, or last follow-up, whichever occurred first. The data cutoff for survival analysis was October 2024.

### 2.5 Statistical analysis

**Figure 1:**
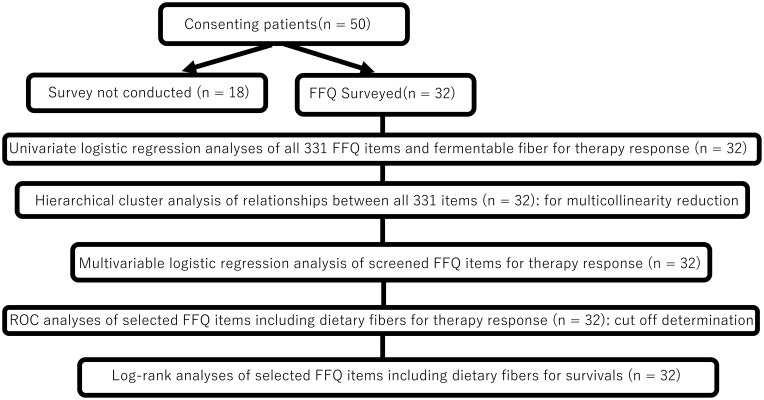
Statistical analysis flowchart. FFQ, food frequency questionnaire; ROC, receiver operating characteristicc (ROC) curves.

Flowchart of statistical analysis process of this study is shown in **Figure 1**. Complete data were available without missing data for all variables in the 32 patients. All analyses were performed using R software (version 4.3.1; the R Foundation for Statistical Computing). The 331 dietary items (variables) were first screened for their association with ICI response. After the variables were normalized into z-scores (as a mean of 0 and a standard deviation of 1), univariate logistic regression (using binary logit model) was applied for each variable with “non-objective response” (SD/PD *vs* CR/PR) and “non-disease control” (PD *vs* CR/PR/SD) as the outcome. To validate, separate univariate logistic regression (using binary logit model) was also applied for each variable, which was adjusted for energy (per kilo calory) and converted to z-score. Variables with P < 0.10 in the univariate analyses were considered candidates. To avoid multicollinearity among closely related foods/nutrients, hierarchical cluster analysis on the 331 variables was conducted using Ward’s method and the squared Euclidean distance using the “ComplexHeatmap” package version 2.18.0 in R (22). If two candidate variables clustered closely (within 10 items of each other on the dendrogram), only one representative was retained from that cluster. The remaining candidates were converted to z-scores and entered into regression model using stepwise backward selection to identify the independent dietary predictors of “non-objective response” to identify the independent dietary predictors of non-objective response. For the survival endpoints (OS and PFS), Kaplan–Meier curves were generated and compared by the log-rank test. Hazard ratios (HRs) were estimated using Cox proportional hazards models. Exploratory analyses were also performed using receiver operating characteristic (ROC) curves to determine the optimal intake cutoff values (e.g., for fiber or acetic acid) to stratify the outcomes. All tests were two-sided, and P < 0.05 was considered significant.

## 3 Results

### 3.1 Patient characteristics and survival

**Figure 2:**
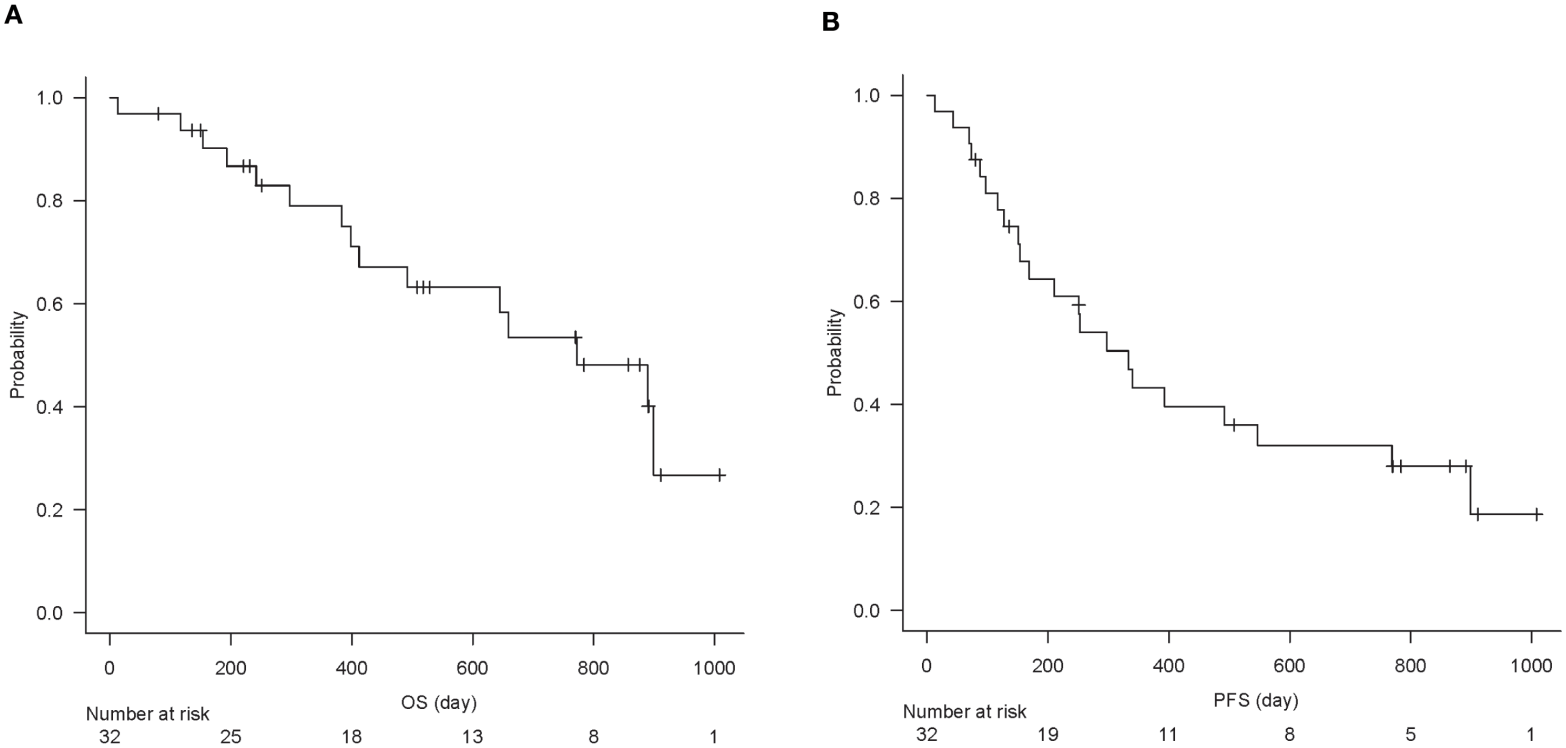
Kaplan–Meier curve of overall survival **(A)** and progression-free survival **(B)** of all analyzed patients (n = 32).

The baseline characteristics of the 32 patients are summarized in **Table 1**. In brief, the cohort predominantly included men (75%) with a median age of 71.5 years. At a median follow-up of 16.4 months (range, 13–1008 days), the median OS was 772 days, and the median PFS was 333 days (**Figures 2A, B**). The best overall responses to ICI therapy were CR in 3 patients, PR in 8, SD in 13, and PD in 8 patients (**Table 1**). The ORR (CR + PR) was 34.4%, and the DCR (CR + PR + SD) was 75.0%.

### 3.2 Dietary factors associated with ICI response

Of the 331 dietary items analyzed, 13 items showed a nominal association with objective response in the univariate logistic regression (P < 0.10), including 3 items (mayonnaise, bacon, and acetic acid (vinegar), P < 0.05) for higher odds of “non-objective response” with low intake (**Table 2**, **Supplementary Table S1**). Seven items showed a nominal association with disease control (P < 0.1, **Supplementary Table S2**). No other food or nutrient met the screening criterion, including total dietary fiber (“non-objective response”, P = 0.44; “non-disease control”, P = 0.85) or fiber subtypes (water-soluble or insoluble; P > 0.3) (**Supplementary Table S1, S2**).

**Table 2:** Odds ratios from the univariate logistic regression analyses of food or nutrition items (converted into z-scores) from the food frequency questionnaire (FFQ), including P < 0.1 and dietary fibers, for the risk of non-objective response, ranked according to P value.

Food or nutrition items	Odds ratio	95% CI	P value
Mayonnaise	0.327	0.110–0.750	0.018
Bacon	0.265	0.073–0.689	0.019
Acetic acid	0.352	0.123–0.798	0.024
Sauce	0.393	0.126–0.889	0.050
Hijiki seaweed	0.381	0.117–0.886	0.056
18:1 n-9 oleic acid	0.454	0.173–0.983	0.066
Salad dressing	0.479	0.201–1.026	0.070
Margarine	0.321	0.064–0.853	0.075
Stir-fried chicken	0.382	0.108–0.924	0.078
Soba (buckwheat noodles)	5.083	1.234–45.405	0.079
Wiener sausage	0.512	0.225–1.072	0.085
Pork soup	0.297	0.054–0.848	0.086
18:1 n-7 cis-vaccenic acid	0.478	0.181–1.032	0.086
Water-soluble dietary fiber	1.408	0.662–3.359	0.396
Total amount of dietary fiber	1.364	0.642–3.269	0.441
Insoluble dietary fiber	1.339	0.631–3.234	0.469

Results of the 331 food or nutrition items from the FFQ are listed in **Supplementary Table S1**.

**Figure 3:**
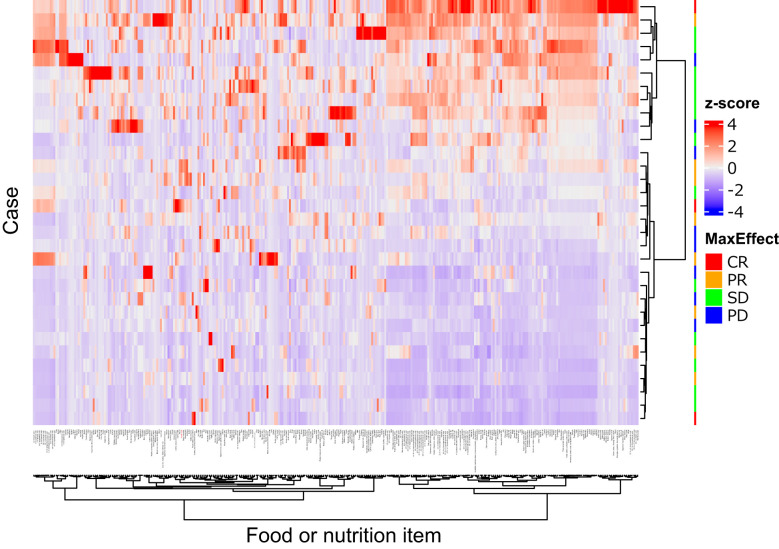
Heatmap of 331 food or nutrition items. Quantity of the items were standardized as z-score (mean = 0.0, standard division = 1.0), and illustrates by the color. The items and cases were clustered by the squared Euclidean distance and Ward’s method, showing as dendrograms. Acetic acid (red colored) and mayonnaise were closely clustered. This figure was drawn using the “ComplexHeatmap” package version 2.18.0 in R.

**Figure 3** illustrates the clustering of dietary variables. Of the 13 candidate items for “non-objective response”, 11 items were closely clustered (4 subclusters: mayonnaise, acetic acid, sauce, hijiki seaweed, and salad dressing; margarine and wiener sausage; stir-fried chicken and pork soup; 18:1 n-9 oleic acid and 18:1 n-7 cis-vaccenic acid), suggesting that these items may provide overlapping information. Half of the patients had no bacon intake, indicating a skewed distribution. Therefore, 9 items (to avoid multicollinearity) and bacon (due to many zero values) were excluded. In the multivariable analysis with stepwise backward selection included 5 items (acetic acid, 18:1 n-9 oleic acid, margarine, stir-fried chicken, and buckwheat noodles), only acetic acid remained as a variable for “non-objective response”. Total food intake (total grams), total dietary fiber, and acetic acid intake were included as predictors in the multivariable logistic model. This model showed a significant overall association (chi-squared test, P = 0.03) with the risk of ICI “non-objective response.” Importantly, acetic acid intake emerged as an independent predictor: patients with higher vinegar consumption had significantly lower odds of “non-objective response” (P = 0.017) (**Table 3**). In other words, even a modest increase in daily vinegar intake was associated with a markedly improved likelihood of achieving an objective response, independent of fiber intake and total food amount.

**Table 3:** Result of the multivariable logistic regression analysis for the risk of a non-objective response (z-score normalized variables).

Food or nutrition items	Odds	95% CI	P value
(Intercept)	2.140	0.9070–5.060	0.0823
Total amount of dietary fiber	2.170	0.4940–9.500	0.3050
Gross weight	0.907	0.2330–3.530	0.8880
Acetic acid	0.276	0.0952–0.798	0.0174

Likelihood-ratio chi-squared test (P = 0.03183); Akaike information criterion = 40.37.

Of 7 candidate items for “non-disease control”, 3 items were excluded due to clustering (2 subclusters: 16:1 palmitoleic acid and 17:1 heptadecenoic acid; meat, 18:1_n-7 cis-vaccenic acid, and 18:2_n-9 oleic acid), 4 items (taro, 16:1palmitoleic acid, 18:1 n-9 oleic acid, and wiener sausage) were excluded with stepwise backward selection. No item remained as a variable for “non-disease control”.

Separate univariate logistic regression analyses of the 331 items adjusted for energy showed that 35 items including acetic acid and fatty acids were nominally associated with “non-objective response” (**Supplementary Table S3**), and that 22 items were nominally associated with “non-disease control” (**Supplementary Table S4**). In the multivariate analysis, no items adjusted for energy were significantly associated with “non-objective response” independently of acetic acid.

Stratified analyses of univariate logistic regression among gastric and esophageal patients (n = 22) showed that no items were significantly associated with “non-objective response” nor “non-disease control” (P ≥ 0.05, data not shown).

### 3.3 Dietary fiber intake and outcomes

**Figure 4:**
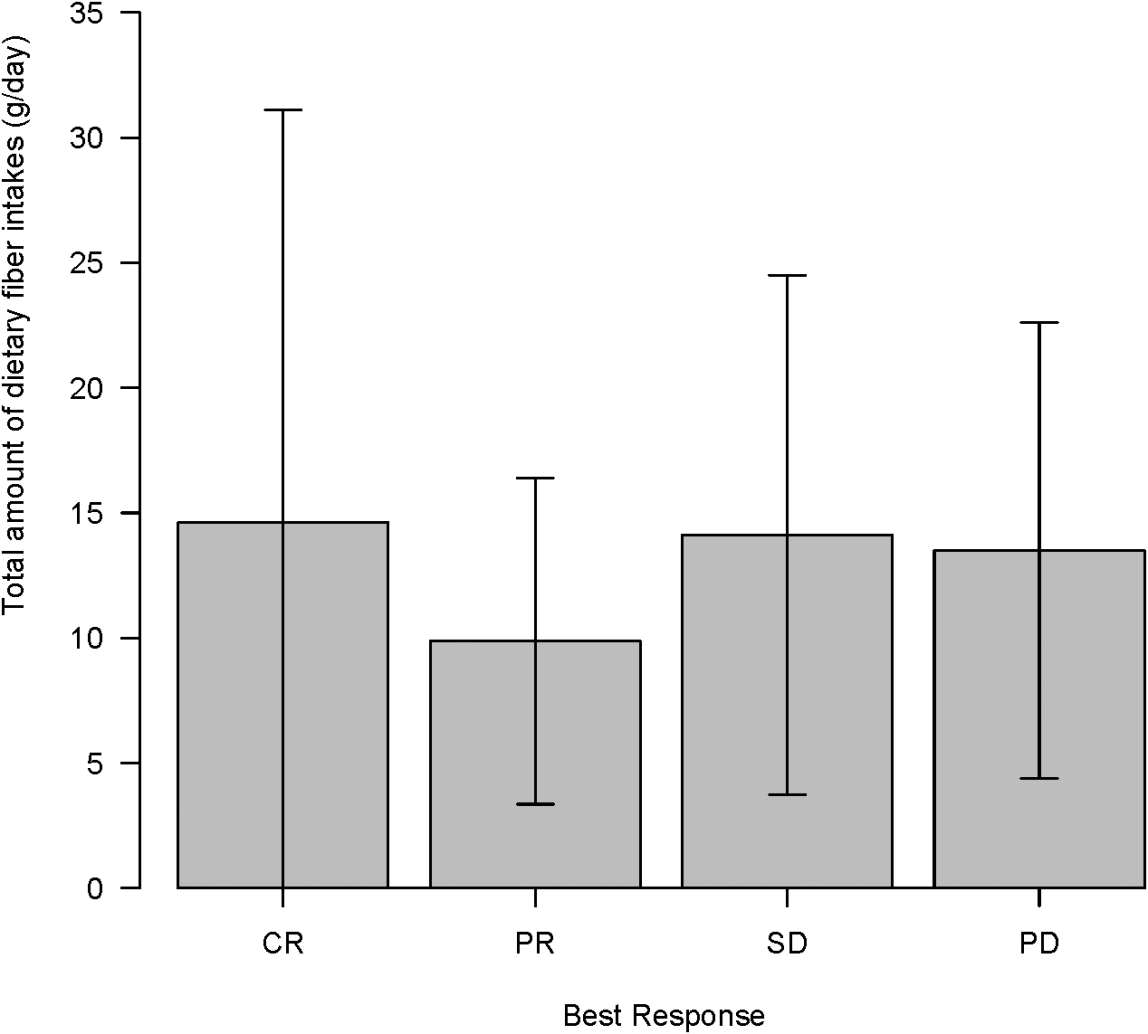
Total amount of dietary fiber intake (g/day) according to immune checkpoint inhibitor maximal response (complete response (CR)/partial response (PR)/stable disease (SD)/progressive disease (PD)) in clinical course.

In our cohort, the median total dietary fiber intake was 8.4 g/day (mean 13.0 ± standard deviation 9.5 g/day). Only 8 of 32 patients (25%) took ≥20 g/day of fiber (a threshold corresponding to that used in the Western study (6). The outcomes were compared between patients with high and low-fiber intake. No significant difference in survival was noted: the median OS was 772 days in the ≥20 g/day group compared with 659 days in the <20 g/day group (log-rank, P = 0.48), and the median PFS was 293 *vs*. 340 days (P = 0.34). A lower fiber cutoff (11.1 g/day) determined by ROC analysis for objective response was also explored, which roughly corresponded to the median fiber intake in a large Japanese cohort (23). Similarly, the OS did not show a significant difference (median, 772 days for ≥11.1 g *vs*. 889 days for <11.1 g, P = 0.80), nor did PFS (297 *vs*. 492 days, P = 0.20) (**Supplementary Figure S1**, **S2**). Consistently, when fiber intake was analyzed as a continuous variable, no association with OS or PFS was observed. Furthermore, patients who responded to ICI (CR/PR) had similar fiber intake (mean, 12–15 g/day) as those who did not respond (SD/PD) (**Figure 4**). No significant differences in the mean total fiber intake were found among the response groups (CR *vs* PR *vs* SD *vs* PD, P = 0.82). The subanalyzes of fiber subtypes did not show an association with either soluble (2.5–3.5 g/day in all groups, P = 0.88) or insoluble (7–11 g/day, P = 0.78) fiber on ICI response.

Then, the subset of fiber most relevant to colonic fermentation (i.e., microbiota-accessible carbohydrates) was examined. The analysis using the estimated fermentable fiber from grains (averaged 10 g/day) similarly did not show a significant association with ICI response or survival. Patients who achieved “objective response” had comparable fermentable fiber intake to those with “non-objective response” (mean, 9.2 *vs* 11.1 g/day, P = 0.79 by the Mann–Whitney test), and fermentable fiber was not a significant predictor of “objective response” (univariate odds ratio, 1.08, P = 0.43) or disease control (odds ratio, 0.82, P = 0.34). The use of an optimal cutoff (11.0 g/day) for fermentable fiber did not stratify OS (median, 645 for ≥ 11.0 g/day *vs* 889 days for < 11.0 g/day, P = 0.54) or PFS (393 *vs* 251 days, P = 0.45). In summary, in this Japanese cohort, dietary fiber intake—whether total or fermentable—showed no clear relationship with ICI efficacy or patient survival.

### 3.4 Acetic acid (vinegar) intake and outcomes

**Figure 5:**
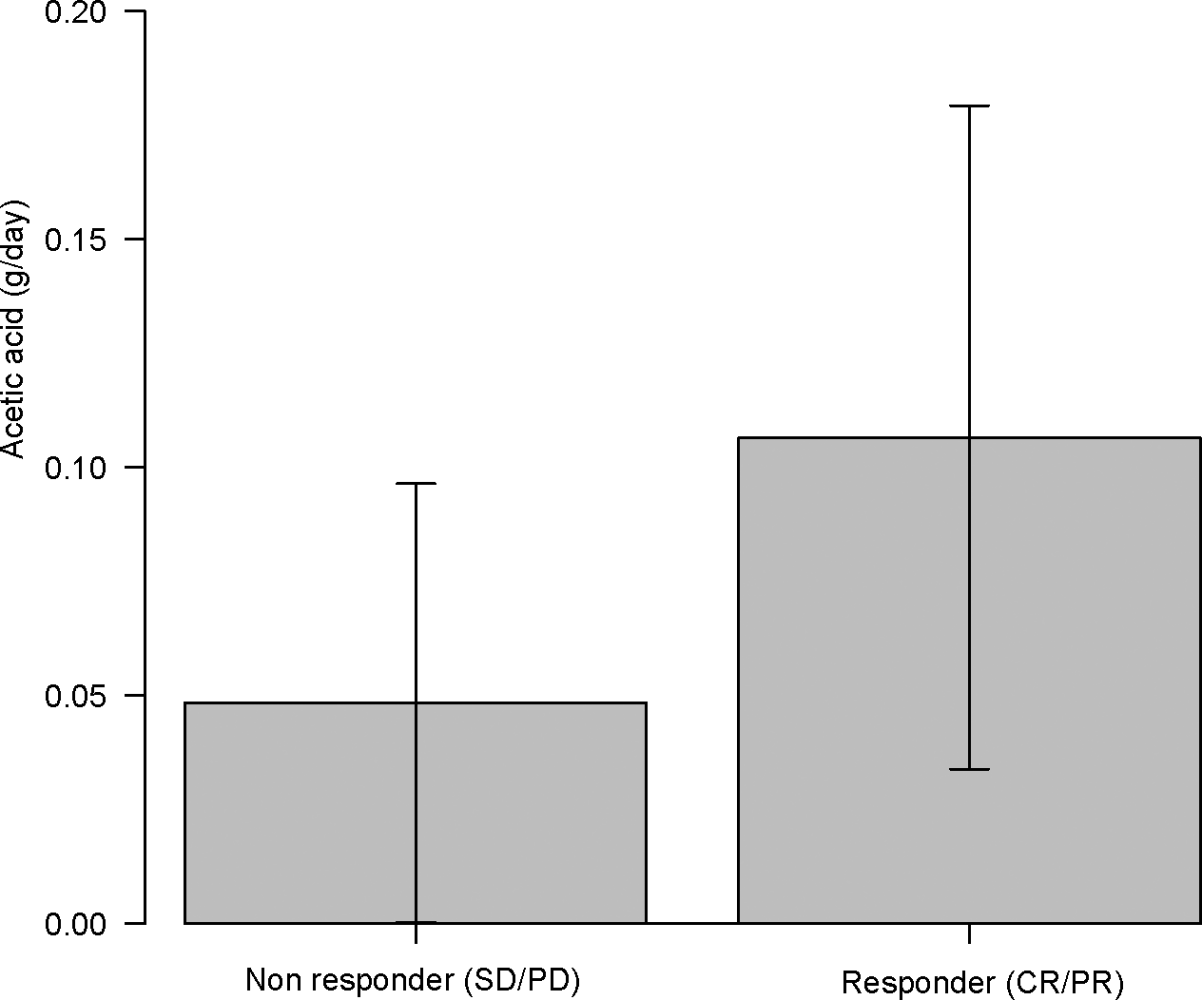
Acetic acid intake (g/day) in immune checkpoint inhibitor (ICI) objective responder (complete response (CR) or partial response (PR)) *vs* non-objective responder (SD or PD). ICI responder had nearly twofold higher vinegar intake than non-responders (P = 0.037).

**Figure 6:**
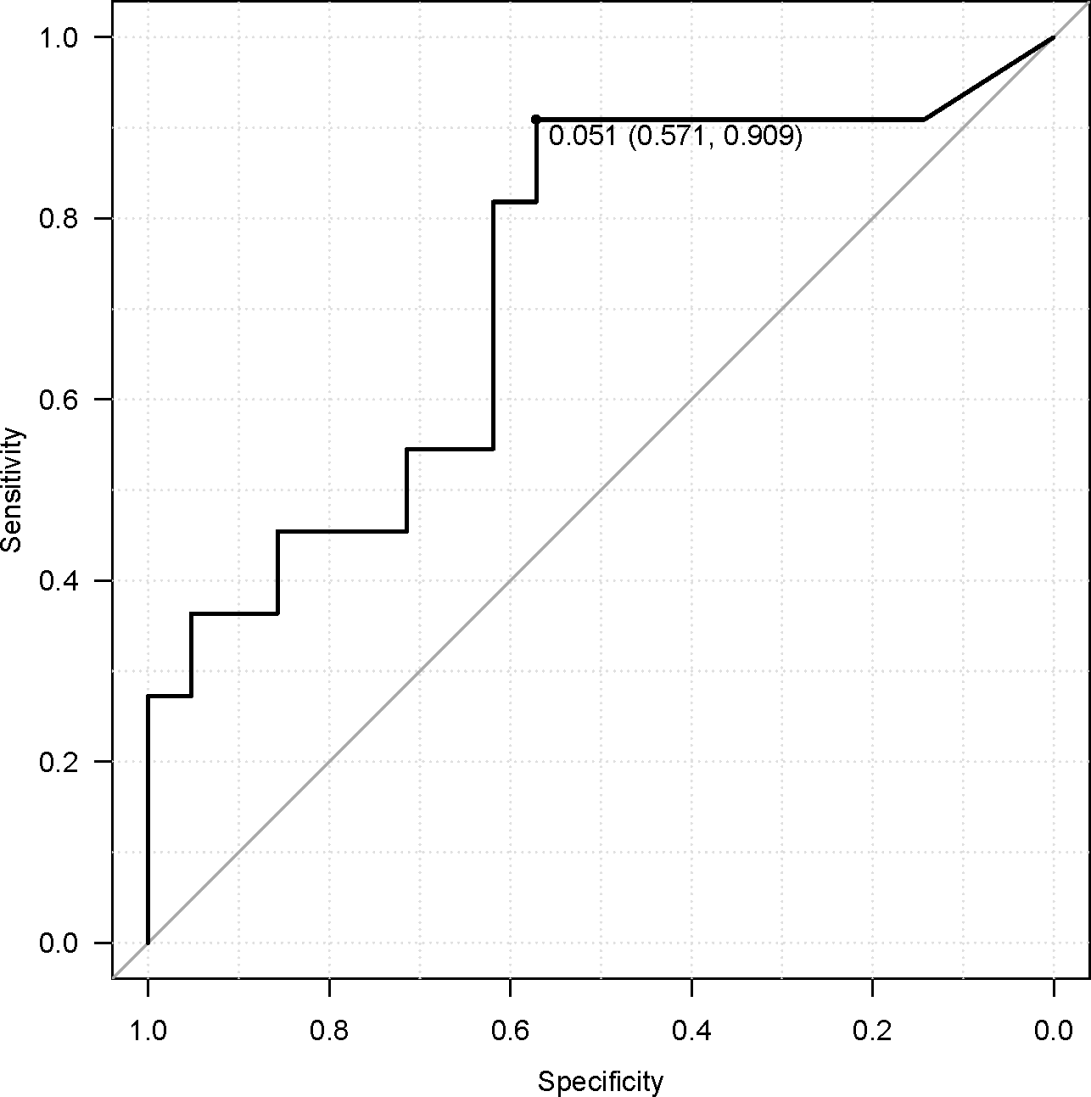
Receiver operating characteristic curve for acetic acid and objective response, showing an optimal cutoff of 0.051 g/day.

**Figure 7:**
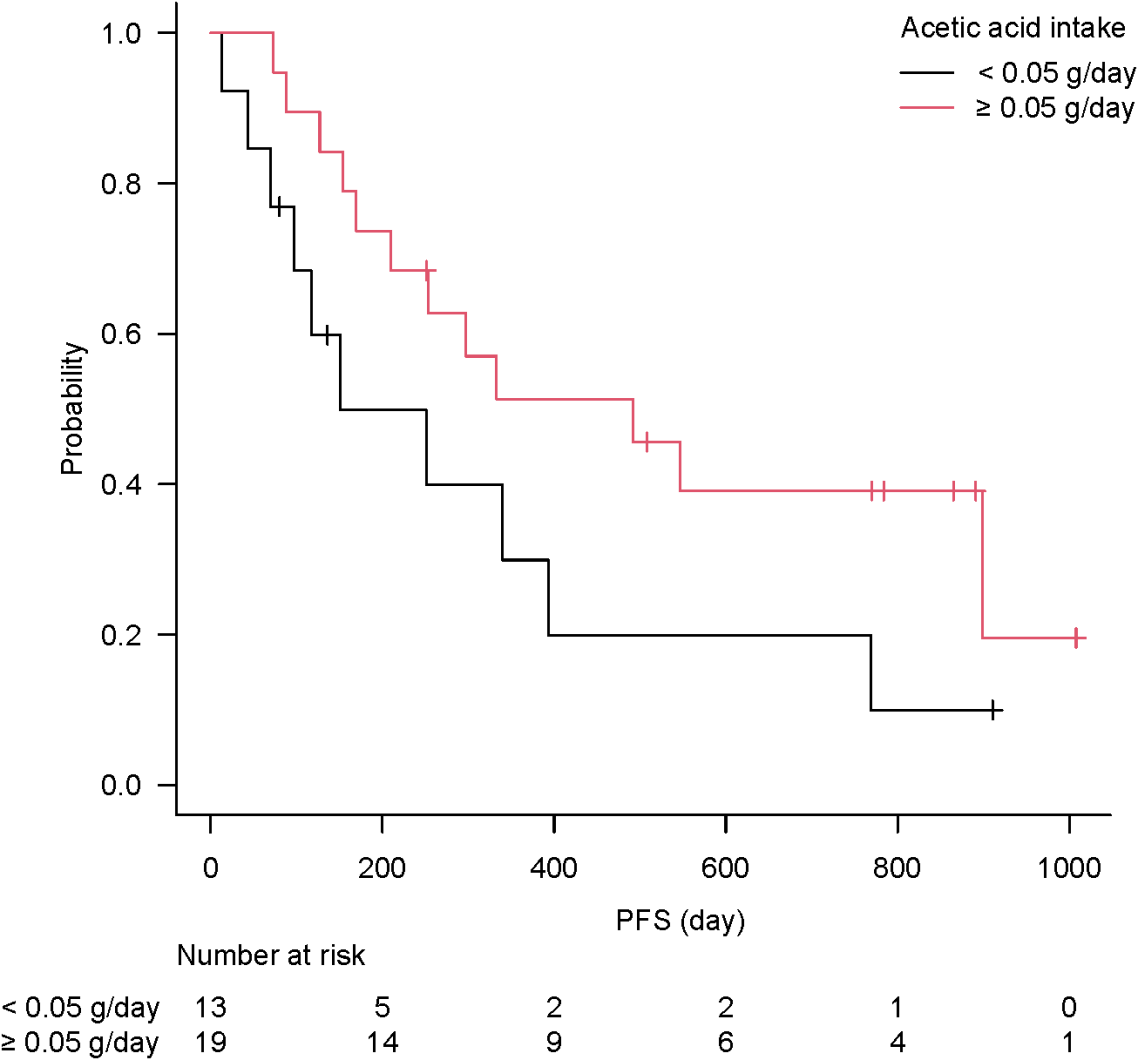
Kaplan–Meier curve of progression-free survival according to vinegar intake. The high vinegar intake group (≥0.05 g/day, n = 19) trended to longer immune checkpoint inhibitor effect than low intake group (<0.05 g/day, n = 13), without significance (P = 0.093).

Acetic acid intake from the diet (primarily from vinegar-containing foods) varied widely among patients, with a median of 0.055 g/day (interquartile range, 0.011–0.100 g/day) and a maximum of 0.222 g/day. Notably, patients who responded to ICI therapy had significantly higher vinegar intake than non-responders (acetic acid intake, 0.107 ± 0.070 g/day for “objective response” *vs* 0.048 ± 0.050 g/day for “non-objective response”; P = 0.037 by the Mann–Whitney test). This nearly twofold difference is illustrated in **Figure 5**. When stratified by response category, median vinegar intake tended to be the highest in the CR group and the lowest in the SD group; however, the differences among the four groups did not reach significance (P = 0.17 by the Kruskal–Wallis test). Using ROC analysis, an optimal cutoff of 0.051 g/day of acetic acid was identified for distinguishing objective responders from non-objective responders (**Figure 6**). Then, the survival outcomes were compared between patients with baseline vinegar intake ≥0.05 g/day (n = 19) and those with <0.05 g/day (n = 13). The high vinegar intake group showed a trend toward longer PFS: the median PFS was 492 days in patients consuming ≥0.05 g compared with 151 days in those consuming less (P = 0.093) (**Figure 7**). Although the PFS difference did not reach the 5% significance level, the HR for progression in the high vinegar intake group was less than half that of the low vinegar intake group (HR 0.49, P = 0.10 in the univariate Cox analysis). No significant difference in OS was observed between the two groups (median OS, 899 *vs* 659 days, P = 0.50). In a Cox model, acetic acid intake as a continuous variable did not show a significant effect on OS or PFS (both P > 0.5). Nevertheless, the overall pattern implies that intake of even relatively small amounts of vinegar from the diet might contribute to the improvement of tumor control and delayed disease progression under ICI therapy.

## 4 Discussion

To the best of our knowledge, this is the first study to identify a positive association between dietary vinegar intake and ICI therapeutic outcomes. In this Japanese patient cohort, higher consumption of vinegar (acetic acid) was associated with improved tumor response to ICIs, whereas dietary fiber intake showed no significant benefit. These findings highlight the importance of considering regional diet and microbiota differences when evaluating nutritional factors in cancer immunotherapy.

Our hierarchical cluster analysis showed no proximity between vinegar intake and fermented foods intake. This suggests that the effect of vinegar is not a confounding factor of general healthy nutritional behavior. The potential immunological effect of vinegar has only recently begun to gain attention (24). Vinegar has a long history as a folk remedy—dating back to Hippocrates in ancient Greece—being used to treat ailments and clean wounds (25, 26). Contemporary studies are now validating some of the health benefits of vinegar, including its anti-inflammatory and metabolic effects (24, 26). For example, a 2024 study demonstrated that hawthorn vinegar could enhance intestinal immunity and improve general health in an animal model (24). However, the mechanisms by which dietary vinegar might influence anticancer immunity are not fully understood. Broadly, two routes have been hypothesized: (1) indirect effects through modulation of the gut microbiota and its metabolites (25) and (2) direct systemic effects of absorbed acetate on host cells and signaling pathways (27, 28).

1. Microbiota-mediated mechanisms: Short-chain fatty acids (SCFAs) produced by gut bacteria, such as acetate, can modulate immune cell function (29). Acetate, propionate, and butyrate serve as important microbial metabolites that influence both innate and adaptive immunity (13). In particular, acetate enhances IgA production in the colon and affects T-cell differentiation (30). Takeuchi et al. reported that increasing colonic acetate levels increased commensal-specific IgA responses and altered CD4^+^ T-cell activity in mice (30). In patients with cancer on immunotherapy, Nomura et al. found that those with higher fecal concentrations of SCFAs (including acetic acid) had significantly longer PFS than those with low concentrations (31). Thus, acetate in the gut environment may contribute to antitumor immunity, for example, by promoting effector T cells or regulating inflammatory signals.

Could higher dietary intake of vinegar lead to increased colonic acetate levels? Theoretically, ingesting vinegar (acetic acid) might enrich acetate in the colon to fuel these immune-beneficial effects. However, physiological studies have indicated that orally consumed acetate is mostly absorbed in the upper gastrointestinal tract and may not reach the large intestine in substantial amounts (32–35). Sugiyama et al. showed that acetate is rapidly absorbed in the stomach and jejunum (32). In a pig model, portal blood levels of acetic acid rose within 10 min of vinegar ingestion and nearly returned to baseline by 30 min, suggesting that little acetic acid survives to enter the colon (36). Thus, a direct increase of luminal acetate in the colon from drinking vinegar is unlikely. Nonetheless, vinegar could still indirectly affect the composition of the gut microbiota. Recently, Xia et al. reported that consuming traditional aged vinegar altered the gut microbiota in mice, notably increasing beneficial taxa such as Akkermansia (phylum Verrucomicrobia), and reduced intestinal inflammation (37). Another study found that supplementation with Monascus (red yeast) vinegar altered the gut microbiota profile in hyperlipidemic rats, accompanying improvements in lipid metabolism and inflammation (38). Vinegar’s microbial effects were speculated to augment bacterial functions such as oxalate degradation (39). Despite the lack of concrete evidence, regular vinegar intake may subtly shift the gut microbiome toward a community that produces more endogenous acetate or other SCFAs, thereby indirectly bolstering antitumor immunity. Future studies should directly examine the gut microbiome changes in patients consuming vinegar.

2. Systemic mechanisms of vinegar (acetic acid): Acetate readily enters the circulation and can act on various host organs (27, 28, 40). Maslowski et al. (2009) provided a key insight by showing that acetate signaling promoted apoptosis of neutrophils and ameliorated colitis in mice through the GPR43 receptor (41). This indicates that circulating SCFAs can influence immune cell turnover and inflammation resolution. In humans, vinegar consumption confers metabolic benefits such as reducing blood pressure and improving glycemic control (26). A meta-analysis by Shahinfar et al. concluded that vinegar intake produces a dose-dependent reduction in systolic blood pressure (42). It was proposed that acetate suppresses the renin–angiotensin system as demonstrated in spontaneously hypertensive rats (40). Acetate also activates AMP-activated protein kinase (AMPK) and PPARα, leading to increased fatty acid oxidation and reduced body fat in mice (27, 43). In the present study, higher vinegar intake might reflect a diet that includes more fermented or pickled products, which could be linked with an overall healthier metabolic profile (28), potentially supporting better immune function. Okura et al. (44) reported that feeding rats a type of brown rice vinegar enhanced their humoral immune responses (high serum levels of IgA and IgM). Although the exact pathways remain unclear, these findings collectively suggest that acetate can modulate immune and metabolic pathways systemically, independent of the gut microbiota. For cancer immunotherapy, systemic factors such as improved metabolic health or reduced inflammatory tone could translate into a more robust antitumor immune response (13). It is intriguing that mayonnaise (which contains vinegar and fatty acids) also showed a positive association with the ICI response in our univariate screening. One speculation is that SCFAs or other compounds present in these foods might subtly prime the immune system or tumor microenvironment to enhance the responsiveness to ICIs.

In contrast, the inefficacy of dietary fiber in our Japanese cohort prompts the consideration of population differences. Spencer et al. (6) reported that high fiber intake correlates with better ICI outcomes in Western populations, likely by enriching SCFA-producing gut bacteria (13). Why did we not see this in Japan? An obvious reason is that the fiber intake in our patients (median 8 g/day) was substantially lower than in those Western studies, where the median intake was >20 g/day (45). Indeed, the majority of Japanese individuals consume less fiber than the Western-recommended levels. A large Japanese cohort study noted that 80% of the participants had fiber intake <16 g/day (23). This general low-fiber diet could lead to a chronically different gut microbiota. Sonnenburg et al. (46) demonstrated that sustained low-fiber diets over multiple generations of mice caused the extinction of certain gut bacterial species, which could not be fully rescued even after reintroducing fiber. Extrapolating to humans, the contemporarily lower fiber intake in Japan (despite a vegetable-rich diet, the reliance on refined rice as a staple yields lower total fiber) might indicate that Japanese patients start with a gut microbiota of lower diversity and fewer fiber-fermenting bacteria. High microbiota diversity has shown a correlation with better ICI responses; thus, a low-diversity microbiome (as might result from chronically low fiber intake) could be less able to mediate fiber’s benefits on immunity (47). In the present study, even patients who managed to consume ≥20 g/day of fiber did not show improved outcomes, possibly because their microbiome was not primed to convert that fiber into beneficial metabolites. Another factor is the difference in the fiber definition and measurement. Until recently, Japanese food composition tables did not count resistant starch and some oligosaccharides as dietary fiber (17). Thus, a Japanese “20 g” of fiber may actually contain less fermentable substrate than an American “20 g” of fiber. We attempted to account for this by estimating the fermentable fiber but still found no association with the outcomes. It may be that once fiber intake is below a certain threshold (e.g., <15 g/day), its influence on the microbiome and immunity is minimal. In summary, our findings indicate that simply increasing fiber intake did not yield the same immunotherapeutic benefit in a low-fiber–adapted population. Instead, more comprehensive or long-term interventions, such as gradually boosting fiber over generations or directly supplementing beneficial microbes, might be required.

This study has several limitations. First, the sample size was relatively small (n = 32), which limits the statistical power, particularly for survival analyses. Age- and sex-based distribution of the study population was not representative of the broader cancer cohort, limiting generalizability of the results. Variables in multivariable logistic regression were screened using univariate logistic regression without multiple comparison correction. The findings in the presenting study should be interpreted as exploratory and preliminary with great caution. Future studies with larger, more diverse populations are needed to validate these preliminary findings. Second, dietary intake was assessed by FFQ, which is subject to recall bias and provides semiquantitative estimates. The FFQ has been validated using 12-day weighed food records by the developing group; dietary fibers correlated adequately (15); however, the percentage of acetic acid differences are large (−50% to −70%) (16). The FFQ is better at ranking individuals than determining absolute intake; therefore, the intake values should not be taken as precise recommendations. Third, our estimation of fermentable fiber was indirect and did not capture all possible microbiota-accessible carbohydrates (for instance, fiber from fruits, vegetables, and seaweed that might also be fermentable were not separately quantified). No established method currently exists to comprehensively measure fermentable fiber intake in Japan. Fourth, patients’ gut microbiomes were not analyzed. Therefore, microbial mechanisms can be only speculated. The present study cannot explain why the ICI responses were associated with vinegar intake and not dietary fiber intake in the Japanese cohort; concurrent microbiome profiling would strengthen future research. Lastly, although we found an association with vinegar intake, vinegar is still a proxy for some other dietary or lifestyle factors. For example, vinegar intake might correlate with the consumption of certain traditional Japanese dishes or with health-conscious behavior. Controlled intervention studies are needed to confirm the causal effect of vinegar on ICI outcomes.

Despite these limitations, our findings raise interesting possibilities for precision nutrition in cancer immunotherapy. Given the difference in diet–microbiome–immune interactions across regions, the “optimal” diet to support ICI therapy may need to be tailored to local habits. In Western populations, a high-fiber diet is emphasized; however, in Japan, simply advising more fiber might not be effective. Instead, other dietary modifications—such as encouraging fermented foods or vinegar (which are familiar in the Japanese diet)—could be more beneficial adjuncts to therapy. Ultimately, personalized nutritional interventions, designed with consideration of an individual’s microbiota and cultural diet, should be explored as a way to improve ICI efficacy. For instance, a prospective trial could test whether adding a small amount of vinegar (e.g., a tablespoon daily; 15 g/tablespoon, equivalent to acetic acid 750 mg as rice vinegar or apple cider vinegar) (48, 49) to the diet of patients on ICIs improves outcomes compared with a control diet. The safety of drinking vinegar containing 750 mg of acetic acid per 100 mL was also reported (50). Likewise, investigating microbiome changes in such trials would help clarify the mechanism. As the field of immuno-nutrition evolves, what constitutes a “favorable food” during ICI therapy will likely differ between a Japanese patient and a Western patient, and this study underscores the importance of this context-specific approach.

## 5 Conclusion

The findings of this study imply that in Japanese patients undergoing ICI therapy, higher vinegar (acetic acid) intake may contribute to a better therapeutic response, whereas dietary fiber intake (at typical Japanese levels) appears to have a limited effect on outcomes. These results underscore that dietary recommendations to enhance ICI efficacy should not adopt a “one-size-fits-all” model but consider regional dietary habits and microbiota profiles. Nutritional interventions that are effective in Western populations may need adaptation in Japan. Integrating modest vinegar consumption into the diet is a feasible approach that could potentially improve ICI responses in Japanese patients; however, further prospective studies are warranted to confirm this benefit. Ultimately, tailoring dietary guidance to individual and ethnic differences—a form of precision nutrition—will be important for optimizing cancer immunotherapy results.

## Data Availability

The original contributions presented in the study are included in the article/**Supplementary Material**. Further inquiries can be directed to the corresponding author.
