# Supplementary material for: Vinegar intake in patients undergoing immune checkpoint inhibitor therapy: food frequency questionnaire study
Source: Front Immunol. 2025 Dec 11;16:1640603. doi: 10.3389/fimmu.2025.1640603 (PMC12738812; doi:10.3389/fimmu.2025.1640603)
Supplement: Supplementary file 1 [file DataSheet1.docx]

Supplementary Material

# Supplementary Figures and Tables

## Supplementary Figures

**Supplementary Figure S1.** Kaplan–Meier curve of overall survival according to total dietary fiber intake with the cutoff value (11.1 g/day) determined by ROC analysis for objective response. Significant differences were not shown in OS (P = 0.80).

**Supplementary Figure S2.** Kaplan–Meier curve of progression free survival according to total dietary fiber intake with the cutoff value (11.1 g/day) determined by ROC analysis for objective response. Significant differences were not shown in PFS (P = 0.20).

## Supplementary Table

**Table S1.** Odds ratios of 331 food or nutrition items (converted into z-scores) in the univariate logistic regression analyses for the risk of non-objective response ranked by P value

| **Food or nutrition items** | **Odds ratio** | **95% CI (low)** |  | **95% CI (high)** | **p value** |
| --- | --- | --- | --- | --- | --- |
| Mayonnaise | 0.327 | 0.110 | - | 0.750 | 0.018 |
| Bacon | 0.265 | 0.073 | - | 0.689 | 0.019 |
| Acetic acid | 0.352 | 0.123 | - | 0.798 | 0.024 |
| Sauce | 0.393 | 0.126 | - | 0.889 | 0.050 |
| Hijiki seaweed | 0.381 | 0.117 | - | 0.886 | 0.056 |
| 18:1 n-9 oleic acid | 0.454 | 0.173 | - | 0.983 | 0.066 |
| Salad dressing | 0.479 | 0.201 | - | 1.026 | 0.070 |
| Margarine | 0.321 | 0.064 | - | 0.853 | 0.075 |
| Stir-fried chicken | 0.382 | 0.108 | - | 0.924 | 0.078 |
| Soba (buckwheat noodles) | 5.083 | 1.234 | - | 45.405 | 0.079 |
| Wiener sausage | 0.512 | 0.225 | - | 1.072 | 0.085 |
| Pork soup | 0.297 | 0.054 | - | 0.848 | 0.086 |
| 18:1 n-7 cis-vaccenic acid | 0.478 | 0.181 | - | 1.032 | 0.086 |
| Ketchup | 0.507 | 0.194 | - | 1.076 | 0.103 |
| Nori seaweed | 2.435 | 0.979 | - | 9.327 | 0.104 |
| Oils and fats | 0.500 | 0.172 | - | 1.083 | 0.119 |
| Boiled pork | 0.262 | 0.034 | - | 0.908 | 0.119 |
| Green onion, Wakegi tree onion | 0.028 | 0.000 | - | 0.806 | 0.119 |
| Japanese tea (made from leaves) | 0.515 | 0.189 | - | 1.103 | 0.123 |
| 20:2 n-6 icosadienoic acid | 0.471 | 0.150 | - | 1.065 | 0.125 |
| Boiled tofu | 2.191 | 0.913 | - | 7.480 | 0.129 |
| Meat | 0.559 | 0.217 | - | 1.185 | 0.161 |
| Carbonated drinks | 4.226 | 1.081 | - | 86.948 | 0.162 |
| Theobromine | 2.229 | 0.879 | - | 10.778 | 0.178 |
| Polyphenol | 2.229 | 0.879 | - | 10.778 | 0.178 |
| Chocolate | 2.229 | 0.879 | - | 10.778 | 0.178 |
| Green asparagus | 2.387 | 0.885 | - | 11.708 | 0.179 |
| Taro | 2.003 | 0.843 | - | 7.329 | 0.182 |
| 22:4 n-6 docosatetraenoic acid | 0.520 | 0.160 | - | 1.161 | 0.184 |
| Alcoholic drinks | 2.597 | 0.876 | - | 14.544 | 0.184 |
| Squid | 2.774 | 0.916 | - | 18.986 | 0.185 |
| α-carotene | 1.908 | 0.826 | - | 6.150 | 0.186 |
| Tofu | 133.428 | 1.205 | - | 1299546.985 | 0.188 |
| Bisket | 1.995 | 0.834 | - | 7.343 | 0.191 |
| Olive oil | 2.531 | 0.893 | - | 17.119 | 0.193 |
| 16:1 palmitoleic acid | 0.623 | 0.274 | - | 1.302 | 0.216 |
| Jam | 0.080 | 0.001 | - | 1.057 | 0.217 |
| Stewed pork | 0.477 | 0.109 | - | 1.142 | 0.217 |
| Somen, Hiyamugi | 2.110 | 0.826 | - | 9.657 | 0.217 |
| 18:1 fatty acid total | 0.623 | 0.269 | - | 1.305 | 0.221 |
| Rapeseed oil and canola oil | 0.601 | 0.195 | - | 1.266 | 0.223 |
| Daikon (Japanese white radish) | 1.776 | 0.783 | - | 5.436 | 0.225 |
| Carrot | 1.762 | 0.779 | - | 5.351 | 0.229 |
| 18:0 stearic acid | 0.631 | 0.279 | - | 1.322 | 0.230 |
| Iodine | 0.637 | 0.290 | - | 1.327 | 0.231 |
| Monounsaturated fatty acids | 0.633 | 0.275 | - | 1.325 | 0.235 |
| Low ham | 0.608 | 0.223 | - | 1.291 | 0.237 |
| Seasonings and spices | 0.635 | 0.272 | - | 1.335 | 0.243 |
| Triacylglycerol equivalent | 0.642 | 0.282 | - | 1.346 | 0.249 |
| 18:3 n-3α-linolenic acid | 0.631 | 0.250 | - | 1.327 | 0.249 |
| Total amount of fatty acids | 0.643 | 0.282 | - | 1.346 | 0.249 |
| 16:0 palmitic acid | 0.647 | 0.290 | - | 1.353 | 0.252 |
| Pickled eggplant | 2.074 | 0.797 | - | 11.586 | 0.255 |
| Fried pork | 0.553 | 0.129 | - | 1.237 | 0.255 |
| Saturated fatty acids | 0.653 | 0.296 | - | 1.366 | 0.260 |
| Lipid | 0.650 | 0.287 | - | 1.362 | 0.261 |
| Sugar, sweeteners | 0.654 | 0.280 | - | 1.360 | 0.261 |
| Beef steak | 2.488 | 0.836 | - | 30.489 | 0.270 |
| 20:3 n-6 icosatrienoic acid | 0.658 | 0.296 | - | 1.377 | 0.270 |
| Stir-fried pork | 0.667 | 0.308 | - | 1.389 | 0.276 |
| Grilled chicken | 0.634 | 0.218 | - | 1.348 | 0.281 |
| Butter | 0.334 | 0.031 | - | 1.099 | 0.284 |
| Canned tuna | 0.670 | 0.302 | - | 1.396 | 0.284 |
| Japanese leek | 0.588 | 0.132 | - | 1.295 | 0.286 |
| Alcohol | 1.686 | 0.740 | - | 5.810 | 0.287 |
| Tara | 0.675 | 0.308 | - | 1.405 | 0.290 |
| 24:0 lignoceric acid | 0.670 | 0.289 | - | 1.402 | 0.293 |
| Available carbohydrates (monosaccharide equivalents) | 1.588 | 0.723 | - | 4.268 | 0.294 |
| Bird liver | 1.899 | 0.756 | - | 9.763 | 0.299 |
| Shrimp | 1.776 | 0.743 | - | 7.916 | 0.306 |
| Snacks (potato chips) | 0.627 | 0.167 | - | 1.355 | 0.308 |
| 22:2 docosadienoic acid | 1.580 | 0.717 | - | 4.556 | 0.309 |
| Salmon | 1.580 | 0.717 | - | 4.556 | 0.309 |
| Fruits | 2.258 | 0.766 | - | 16.865 | 0.311 |
| Chromium | 1.574 | 0.714 | - | 4.416 | 0.312 |
| Oolong tea (cans and plastic bottles) | 0.685 | 0.293 | - | 1.431 | 0.315 |
| 20:0 arachidic acid | 0.685 | 0.302 | - | 1.435 | 0.318 |
| Black tea (cans and plastic bottles) | 0.632 | 0.165 | - | 1.370 | 0.323 |
| Japanese tea (cans and plastic bottles) | 1.763 | 0.732 | - | 8.183 | 0.326 |
| Beer | 3.509 | 0.790 | - | 130.820 | 0.328 |
| Beans | 1.538 | 0.702 | - | 4.201 | 0.329 |
| Pumpkin | 1.981 | 0.749 | - | 12.460 | 0.331 |
| Cod roe, Sujiko, and salmon roe | 0.669 | 0.227 | - | 1.422 | 0.331 |
| Organic acid | 2.255 | 0.750 | - | 17.619 | 0.336 |
| Pickled green leaves | 2.118 | 0.754 | - | 19.094 | 0.338 |
| n-6 polyunsaturated fatty acids | 0.693 | 0.293 | - | 1.458 | 0.339 |
| 18:2 n-6 linoleic acid | 0.696 | 0.295 | - | 1.464 | 0.344 |
| 14:0 myristic acid | 0.705 | 0.328 | - | 1.475 | 0.348 |
| 20:4 n-6 arachidonic acid | 0.706 | 0.318 | - | 1.482 | 0.356 |
| Coffee (made from beans) | 0.696 | 0.260 | - | 1.472 | 0.357 |
| Polyunsaturated fatty acids | 0.706 | 0.302 | - | 1.485 | 0.361 |
| Deep-fried thick tofu | 1.574 | 0.693 | - | 5.294 | 0.362 |
| Magnesium | 1.462 | 0.679 | - | 3.701 | 0.365 |
| Fried chicken | 0.703 | 0.276 | - | 1.489 | 0.367 |
| Luxury drinks | 0.717 | 0.320 | - | 1.506 | 0.374 |
| Carbohydrates | 1.445 | 0.673 | - | 3.609 | 0.376 |
| Coffee sugar | 0.721 | 0.313 | - | 1.514 | 0.378 |
| Chive | 0.531 | 0.020 | - | 1.286 | 0.390 |
| Salt cod and salt salmon | 1.576 | 0.684 | - | 6.156 | 0.390 |
| Water-soluble dietary fiber | 1.408 | 0.662 | - | 3.359 | 0.396 |
| Seafood | 1.431 | 0.664 | - | 3.725 | 0.398 |
| Copper | 1.396 | 0.658 | - | 3.238 | 0.400 |
| Boiled chicken | 0.734 | 0.325 | - | 1.544 | 0.401 |
| 17:0 heptadecanoic acid | 0.732 | 0.341 | - | 1.539 | 0.406 |
| Grains | 1.403 | 0.657 | - | 3.404 | 0.407 |
| β-Cryptoxanthin | 1.682 | 0.693 | - | 11.908 | 0.409 |
| Dried fish | 1.409 | 0.657 | - | 3.661 | 0.413 |
| 16:0 isopalmitic acid | 0.740 | 0.347 | - | 1.555 | 0.415 |
| Pickled cucumber | 1.545 | 0.674 | - | 7.328 | 0.416 |
| 17:1 heptadecenoic acid | 0.738 | 0.343 | - | 1.552 | 0.418 |
| 10:0 decanoic acid | 0.744 | 0.348 | - | 1.563 | 0.422 |
| Beef stew | 1.658 | 0.679 | - | 8.766 | 0.422 |
| 20:1 icosenoic acid | 0.743 | 0.339 | - | 1.565 | 0.425 |
| 4:0 butyric acid | 0.745 | 0.349 | - | 1.568 | 0.425 |
| Koya tofu (freeze-dried tofu) | 1.662 | 0.680 | - | 12.663 | 0.430 |
| Mixed oil | 0.731 | 0.232 | - | 1.576 | 0.431 |
| 18:3 n-6 γ-linolenic acid | 1.391 | 0.648 | - | 3.535 | 0.432 |
| Broccoli | 1.555 | 0.666 | - | 6.872 | 0.432 |
| 15:0 pentadecanoic acid | 0.748 | 0.351 | - | 1.574 | 0.435 |
| Grapes | 12.848 | 0.760 | - | 227627.684 | 0.437 |
| 22:0 behenic acid | 0.751 | 0.343 | - | 1.584 | 0.439 |
| Total amount of dietary fiber | 1.364 | 0.642 | - | 3.269 | 0.441 |
| 12:0 lauric acid | 0.753 | 0.338 | - | 1.593 | 0.444 |
| Pickles | 1.373 | 0.641 | - | 3.642 | 0.452 |
| Cabbage | 1.357 | 0.637 | - | 3.351 | 0.455 |
| Onion | 1.456 | 0.651 | - | 5.860 | 0.456 |
| Mandarin orange | 1.508 | 0.657 | - | 8.777 | 0.459 |
| γ-Tocopherol | 0.761 | 0.329 | - | 1.619 | 0.467 |
| Daidzein | 1.365 | 0.635 | - | 3.714 | 0.468 |
| Genistein | 1.363 | 0.635 | - | 3.685 | 0.468 |
| Eel | 0.767 | 0.363 | - | 1.620 | 0.468 |
| Insoluble dietary fiber | 1.339 | 0.631 | - | 3.234 | 0.469 |
| 10:1 decenoic acid | 0.766 | 0.361 | - | 1.617 | 0.470 |
| Yogurt | 1.465 | 0.648 | - | 7.789 | 0.473 |
| n-3 polyunsaturated fatty acids | 0.766 | 0.347 | - | 1.622 | 0.474 |
| Chinese cabbage | 1.356 | 0.632 | - | 3.629 | 0.474 |
| Pickled takuwan (pickled daikon radish) | 1.922 | 0.682 | - | 35.760 | 0.478 |
| Garlic | 1.914 | 0.679 | - | 27.599 | 0.479 |
| Coffee milk | 0.027 | 0.000 | - | 0.966 | 0.479 |
| 15:0 antpentadecanoic acid | 0.771 | 0.364 | - | 1.629 | 0.480 |
| Vitamin B12 | 1.764 | 0.667 | - | 16.649 | 0.483 |
| Banana | 1.678 | 0.657 | - | 18.995 | 0.498 |
| 6:0 hexanoic acid | 0.780 | 0.368 | - | 1.652 | 0.500 |
| 17:0 antheptadecanoic acid | 0.781 | 0.369 | - | 1.653 | 0.502 |
| 8:0 octanoic acid | 0.783 | 0.367 | - | 1.662 | 0.507 |
| 14:1 myristoleic acid | 0.780 | 0.367 | - | 1.646 | 0.507 |
| Citrus | 0.786 | 0.322 | - | 1.708 | 0.513 |
| Vitamin C | 1.317 | 0.616 | - | 3.654 | 0.520 |
| Noodle soup | 1.422 | 0.626 | - | 6.651 | 0.520 |
| Shochu | 1.323 | 0.617 | - | 4.054 | 0.521 |
| Moisture | 1.290 | 0.610 | - | 3.020 | 0.521 |
| Gross weight | 1.288 | 0.609 | - | 2.998 | 0.522 |
| Wakame seaweed | 1.318 | 0.614 | - | 3.720 | 0.524 |
| Enoki mushroom | 1.357 | 0.617 | - | 5.645 | 0.531 |
| Japanese mustard | 0.794 | 0.361 | - | 1.700 | 0.531 |
| Tap water | 1.289 | 0.607 | - | 3.281 | 0.538 |
| Kiwi fruit | 3.682 | 0.704 | - | 14006.386 | 0.538 |
| Japanese mustard spinach | 0.800 | 0.338 | - | 1.749 | 0.543 |
| Vitamin B6 | 1.277 | 0.604 | - | 3.094 | 0.544 |
| Milk | 0.803 | 0.362 | - | 1.737 | 0.547 |
| Calcium | 1.285 | 0.604 | - | 3.443 | 0.550 |
| Cake | 1.288 | 0.603 | - | 3.641 | 0.555 |
| Folic acid | 1.262 | 0.598 | - | 3.025 | 0.560 |
| 22:1 docosenoic acid | 1.255 | 0.596 | - | 2.875 | 0.561 |
| Whitebait | 1.300 | 0.601 | - | 4.193 | 0.565 |
| Iron | 1.250 | 0.593 | - | 2.827 | 0.567 |
| Eggplant | 1.358 | 0.604 | - | 7.902 | 0.573 |
| Ice cream | 1.276 | 0.596 | - | 3.862 | 0.578 |
| Japanese pear | 1.299 | 0.600 | - | 5.737 | 0.579 |
| Stir-fried beef | 0.817 | 0.385 | - | 1.751 | 0.582 |
| Biotin | 1.242 | 0.589 | - | 2.917 | 0.583 |
| Tannin | 0.818 | 0.352 | - | 1.803 | 0.583 |
| Strawberry | 1.582 | 0.627 | - | 66.839 | 0.585 |
| Cheese | 1.244 | 0.590 | - | 3.031 | 0.586 |
| Rice cake | 0.822 | 0.370 | - | 1.797 | 0.592 |
| Nitrate ion | 0.821 | 0.337 | - | 1.826 | 0.592 |
| Egg | 1.229 | 0.583 | - | 2.801 | 0.597 |
| Eggs | 1.229 | 0.583 | - | 2.801 | 0.597 |
| 18:4 n-3 octadecatetraenoic acid | 1.227 | 0.583 | - | 2.800 | 0.599 |
| Molybdenum | 1.232 | 0.584 | - | 2.964 | 0.601 |
| Caffeine | 0.828 | 0.359 | - | 1.837 | 0.605 |
| 20:4 n-3 icosatetraenoic acid | 1.221 | 0.580 | - | 2.799 | 0.609 |
| Vegetable juice | 1.253 | 0.582 | - | 4.246 | 0.617 |
| Selenium | 1.221 | 0.580 | - | 2.963 | 0.617 |
| Pantothenic acid | 1.215 | 0.578 | - | 2.818 | 0.620 |
| Spinach | 1.213 | 0.578 | - | 2.873 | 0.624 |
| Safflower oil | 1.331 | 0.584 | - | NA | 0.627 |
| Potatoes | 1.211 | 0.576 | - | 2.912 | 0.630 |
| Pacific saury | 1.208 | 0.575 | - | 2.827 | 0.631 |
| Melon | 1.263 | 0.578 | - | 6.550 | 0.633 |
| Watermelon | 0.842 | 0.393 | - | 1.845 | 0.637 |
| Proline | 1.207 | 0.572 | - | 3.044 | 0.642 |
| Clams | 1.854 | 0.611 | - | NA | 0.649 |
| Lycopene | 1.209 | 0.572 | - | 3.479 | 0.651 |
| Shungiku (garland chrysanthemum) | 0.281 | 0.000 | - | 1.219 | 0.651 |
| Soup | 1.201 | 0.568 | - | 3.275 | 0.658 |
| Confectionery | 1.198 | 0.569 | - | 3.340 | 0.662 |
| Green bell pepper | 0.854 | 0.385 | - | 1.927 | 0.662 |
| Tomato juice | 1.216 | 0.567 | - | 4.518 | 0.662 |
| 22:6 n-3 docosahexaenoic acid | 1.183 | 0.563 | - | 2.656 | 0.664 |
| Phosphorus | 1.185 | 0.565 | - | 2.800 | 0.666 |
| Coffee (cans, plastic bottles) | 0.856 | 0.380 | - | 1.929 | 0.669 |
| Burdock | 0.855 | 0.409 | - | 1.845 | 0.670 |
| Peach | 1.237 | 0.562 | - | 8.603 | 0.670 |
| Bread | 1.233 | 0.560 | - | 8.631 | 0.675 |
| 7:0 heptanoic acid | 1.185 | 0.564 | - | 3.201 | 0.677 |
| 20:5 n-3 icosapentaenoic acid | 1.175 | 0.560 | - | 2.674 | 0.678 |
| 13:0 tridecanoic acid | 1.184 | 0.563 | - | 3.189 | 0.680 |
| Cystine | 1.176 | 0.560 | - | 2.765 | 0.681 |
| Glutamic acid | 1.175 | 0.559 | - | 2.836 | 0.684 |
| Wasabi | 1.174 | 0.559 | - | 2.841 | 0.687 |
| Other vegetables | 1.172 | 0.559 | - | 2.907 | 0.691 |
| Ammonia | 1.173 | 0.558 | - | 2.916 | 0.691 |
| Vitamin D | 1.167 | 0.556 | - | 2.663 | 0.691 |
| Milks | 1.166 | 0.556 | - | 3.000 | 0.702 |
| 21:5 n-3 henicosapentaenoic acid | 1.160 | 0.553 | - | 2.650 | 0.702 |
| Potato | 1.161 | 0.555 | - | 2.717 | 0.703 |
| Bonito | 1.164 | 0.553 | - | 3.075 | 0.709 |
| α-Tocopherol | 0.872 | 0.411 | - | 1.885 | 0.713 |
| Udon | 1.161 | 0.550 | - | 3.032 | 0.714 |
| 16:2 hexadecadienoic acid | 1.150 | 0.549 | - | 2.620 | 0.718 |
| Vitamin B1 | 0.876 | 0.411 | - | 1.906 | 0.720 |
| Niacin | 0.876 | 0.415 | - | 1.898 | 0.721 |
| Ramen noodles | 0.878 | 0.418 | - | 1.931 | 0.723 |
| Mushrooms | 1.154 | 0.548 | - | 3.115 | 0.726 |
| Black tea (made from leaves) | 0.881 | 0.409 | - | 2.004 | 0.727 |
| 16:4 hexadecatetraenoic acid | 1.145 | 0.546 | - | 2.617 | 0.727 |
| Deep-fried thin tofu | 1.148 | 0.547 | - | 3.206 | 0.735 |
| Tomato | 0.885 | 0.402 | - | 2.055 | 0.736 |
| 16:3 hexadecatrienoic acid | 1.139 | 0.543 | - | 2.618 | 0.737 |
| β-Carotene equivalent | 1.142 | 0.544 | - | 2.893 | 0.740 |
| Shiitake mushroom | 1.137 | 0.543 | - | 2.745 | 0.744 |
| Bean sprouts | 0.887 | 0.412 | - | 1.969 | 0.745 |
| Pork liver | 0.889 | 0.409 | - | 2.040 | 0.746 |
| Vegetables | 1.136 | 0.543 | - | 2.882 | 0.749 |
| Persimmon | 1.140 | 0.541 | - | 3.504 | 0.751 |
| Yellowtail | 0.889 | 0.428 | - | 1.938 | 0.751 |
| Potassium | 1.130 | 0.539 | - | 2.585 | 0.753 |
| Algae | 1.130 | 0.539 | - | 2.704 | 0.755 |
| Spaghetti macaroni | 1.131 | 0.538 | - | 2.840 | 0.757 |
| Coffee (instant) | 0.897 | 0.404 | - | 2.110 | 0.766 |
| Ash | 1.115 | 0.532 | - | 2.513 | 0.778 |
| Yam, Chinese yam | 1.116 | 0.534 | - | 2.759 | 0.780 |
| Phenylalanine | 1.113 | 0.532 | - | 2.554 | 0.781 |
| Peanuts | 0.905 | 0.429 | - | 2.042 | 0.787 |
| Serin | 1.109 | 0.530 | - | 2.531 | 0.788 |
| Seeds and fruits | 0.906 | 0.429 | - | 2.048 | 0.789 |
| Zinc | 1.108 | 0.529 | - | 2.485 | 0.790 |
| Apple | 0.906 | 0.433 | - | 1.978 | 0.790 |
| Energy (kJ) | 1.105 | 0.526 | - | 2.456 | 0.793 |
| Energy (kcal) | 1.105 | 0.526 | - | 2.457 | 0.793 |
| Cholesterol | 0.908 | 0.429 | - | 1.941 | 0.799 |
| vitamin K | 0.911 | 0.435 | - | 1.980 | 0.803 |
| Rice crackers | 1.106 | 0.522 | - | 3.504 | 0.806 |
| Tryptophan | 1.097 | 0.524 | - | 2.488 | 0.811 |
| Cooking salt | 0.916 | 0.427 | - | 2.063 | 0.812 |
| Retinol activity equivalent | 1.098 | 0.522 | - | 2.786 | 0.812 |
| Total aromatic amino acids | 1.095 | 0.524 | - | 2.495 | 0.813 |
| Green and yellow vegetables | 1.093 | 0.525 | - | 2.772 | 0.821 |
| Horse mackerel | 1.085 | 0.520 | - | 2.516 | 0.833 |
| 22:5 n-6 docosapentaenoic acid | 1.083 | 0.517 | - | 2.439 | 0.836 |
| Valin | 1.080 | 0.517 | - | 2.445 | 0.840 |
| Tyrosine | 1.079 | 0.516 | - | 2.444 | 0.843 |
| Protein | 1.079 | 0.516 | - | 2.442 | 0.843 |
| Yakult | 0.929 | 0.446 | - | 2.125 | 0.843 |
| Kamaboko (boiled fish paste) | 1.080 | 0.508 | - | 3.126 | 0.846 |
| Protein by amino acid composition table | 1.077 | 0.515 | - | 2.438 | 0.847 |
| Total sulfur-containing amino acids | 1.074 | 0.514 | - | 2.419 | 0.852 |
| Commercial water | 0.935 | 0.428 | - | 2.281 | 0.855 |
| Amino acid total | 1.073 | 0.513 | - | 2.426 | 0.855 |
| Sweet potato | 1.072 | 0.516 | - | 2.668 | 0.858 |
| Water | 1.069 | 0.509 | - | 2.648 | 0.863 |
| 24:1 tetracosenoic acid | 0.938 | 0.447 | - | 2.044 | 0.864 |
| Green bean | 1.066 | 0.509 | - | 2.713 | 0.869 |
| Leucine | 1.064 | 0.510 | - | 2.402 | 0.871 |
| Isoleucine | 1.064 | 0.510 | - | 2.398 | 0.871 |
| Octopus | 1.066 | 0.507 | - | 2.975 | 0.871 |
| Konjac | 1.060 | 0.509 | - | 2.444 | 0.878 |
| Retinol | 1.059 | 0.502 | - | 2.746 | 0.882 |
| Japanese sake | 1.058 | 0.509 | - | 2.829 | 0.885 |
| Pine | 0.948 | 0.433 | - | 2.400 | 0.885 |
| Glycine | 0.950 | 0.453 | - | 2.083 | 0.890 |
| Aspartic acid | 1.048 | 0.501 | - | 2.331 | 0.902 |
| Sodium | 1.044 | 0.497 | - | 2.320 | 0.909 |
| Manganese | 1.041 | 0.493 | - | 2.284 | 0.915 |
| Satsuma-age (fried fish paste) | 1.041 | 0.501 | - | 2.529 | 0.917 |
| 22:5 n-3 docosapentaenoic acid | 0.962 | 0.459 | - | 2.108 | 0.918 |
| Natto | 1.039 | 0.498 | - | 2.508 | 0.921 |
| Salt equivalent amount | 1.038 | 0.494 | - | 2.303 | 0.921 |
| Miso soup | 1.037 | 0.498 | - | 2.424 | 0.924 |
| β-Carotene | 1.035 | 0.494 | - | 2.515 | 0.928 |
| Sesame | 0.967 | 0.466 | - | 2.210 | 0.929 |
| Threonine | 1.031 | 0.494 | - | 2.297 | 0.936 |
| Arginine | 1.031 | 0.492 | - | 2.284 | 0.937 |
| Vitamin B2 | 1.030 | 0.494 | - | 2.309 | 0.937 |
| Lettuce | 1.028 | 0.494 | - | 2.310 | 0.942 |
| Cucumber | 0.974 | 0.459 | - | 2.414 | 0.944 |
| β-Tocopherol | 1.023 | 0.490 | - | 2.338 | 0.952 |
| Soy milk | 1.023 | 0.495 | - | 2.359 | 0.952 |
| δ-Tocopherol | 0.979 | 0.468 | - | 2.185 | 0.954 |
| Lysine | 0.981 | 0.469 | - | 2.159 | 0.960 |
| Chikuwa (tubular fish-paste) | 1.019 | 0.480 | - | 2.602 | 0.960 |
| Pickled Chinese cabbage | 1.018 | 0.491 | - | 2.333 | 0.962 |
| Sea bream | 1.018 | 0.488 | - | 2.429 | 0.962 |
| Rice | 0.983 | 0.463 | - | 2.123 | 0.965 |
| Methionine | 1.016 | 0.486 | - | 2.255 | 0.967 |
| Fruit drinks (not 100%) | 1.015 | 0.490 | - | 2.572 | 0.968 |
| Alanine | 0.986 | 0.471 | - | 2.175 | 0.969 |
| Japanese sweets | 1.014 | 0.471 | - | 2.770 | 0.971 |
| Shimeji mushroom | 1.012 | 0.486 | - | 2.453 | 0.975 |
| Grilled beef | 0.992 | 0.479 | - | 2.263 | 0.983 |
| Histidine | 1.006 | 0.482 | - | 2.236 | 0.987 |
| Pickled turnips | 1.006 | 0.485 | - | 2.342 | 0.988 |
| Pickled umeboshi (pickled plums) | 0.997 | 0.468 | - | 2.604 | 0.993 |
| Orange juice | 943.491 | 0.000 | - | NA | 0.993 |
| Apple juice | 943.491 | 0.000 | - | NA | 0.993 |
| Tea milk | 0.000 | NA | - | Inf | 0.993 |
| Whiskey | 445.080 | 0.000 | - | NA | 0.994 |
| Wine | 0.047 | NA | - | 1.573E + 36 | 0.994 |
| Black tea sugar | 0.047 | NA | - | 1.573E + 36 | 0.994 |
| Oolong tea (made from leaves) | 0.000 | NA | - | 5.031E + 123 | 0.995 |
| Soybean oil | 16.824 | 0.000 | - | NA | 0.995 |
| Low-fat milk | 3.422E + 15 | 0.000 | - | NA | 0.995 |
| Others | 5882366.223 | 0.000 | - | NA | 0.996 |
| cooking oil | NA | NA |  |  |  |
| 15:1pentadecenoic acid | NA | NA |  |  |  |
| 17:2Heptadecadienoic acid | NA | NA |  |  |  |
| 18:2Octadecadienoic acid | NA | NA |  |  |  |
| 18:3Octadecatrienoic acid | NA | NA |  |  |  |
| 100% grapefruit juice | NA | NA |  |  |  |

NA, not available.

**Table S2.** Odds ratios of 331 food or nutrition items (converted into z-scores) in the univariate logistic regression analyses for the risk of non-disease control ranked by P value

| **Food or nutrition items** | **Odds ratio** | **95% CI (low)** | | **95% CI (high)** | **P value** |
| --- | --- | --- | --- | --- | --- |
| Taro | 2.20643 | 1.01528 | - | 5.37356 | 0.05477 |
| 16:1palmitoleic acid | 0.23065 | 0.03144 | - | 0.84491 | 0.07706 |
| 17:1Heptadecenoic acid | 0.30757 | 0.05933 | - | 0.91525 | 0.07972 |
| 18:1n-9oleic acid | 0.19242 | 0.01833 | - | 0.81412 | 0.08359 |
| meat | 0.07456 | 0.0016 | - | 0.63951 | 0.0849 |
| Wiener sausage | 0.03928 | 0.00027 | - | 0.47204 | 0.08812 |
| 18:1n-7cis-vaccenic acid | 0.20929 | 0.02062 | - | 0.86073 | 0.09262 |
| 20:2 n-6Icosadienoic acid | 0.2027 | 0.0198 | - | 0.92841 | 0.10218 |
| 17:0heptadecanoic acid | 0.39502 | 0.09479 | - | 1.05726 | 0.11397 |
| Chinese cabbage | 1.87418 | 0.86245 | - | 4.56306 | 0.1227 |
| Luxury drinks | 0.32235 | 0.05631 | - | 1.05539 | 0.13291 |
| monounsaturated fatty acids | 0.37716 | 0.07936 | - | 1.09539 | 0.13839 |
| Bonito | 0.22852 | 0.01895 | - | 1.00212 | 0.13869 |
| 20:3 n-6Icosatrienoic acid | 0.40116 | 0.09043 | - | 1.10863 | 0.13954 |
| Japanese tea (cans/plastic bottles) | 0.0957 | 0.00114 | - | 0.85849 | 0.14233 |
| 18:1Total | 0.38388 | 0.08149 | - | 1.10839 | 0.14333 |
| grilled chicken | 0.08764 | 0.00152 | - | 0.91572 | 0.1438 |
| 22:4 n-6docosatetraenoic acid | 0.24531 | 0.02454 | - | 1.07468 | 0.14493 |
| Ramen (noodles) | 0.07387 | 0.00095 | - | 0.76601 | 0.14502 |
| 16:0palmitic acid | 0.42523 | 0.1026 | - | 1.14333 | 0.15112 |
| 14:1myristoleic acid | 0.45997 | 0.12548 | - | 1.16618 | 0.152 |
| Bacon | 0.24684 | 0.01576 | - | 0.9969 | 0.15721 |
| 18:0stearic acid | 0.43227 | 0.10622 | - | 1.15999 | 0.1575 |
| Shiitake mushroom | 1.75964 | 0.80947 | - | 4.25935 | 0.1591 |
| 20:1Icosenoic acid | 0.39717 | 0.08274 | - | 1.14303 | 0.16032 |
| Shochu | 1.69405 | 0.79625 | - | 3.80716 | 0.16419 |
| Stir-fried beef | 0.18676 | 0.00646 | - | 0.92736 | 0.16558 |
| Total amount of fatty acids | 0.44066 | 0.10834 | - | 1.18659 | 0.17038 |
| Triacylglycerol equivalent | 0.44201 | 0.10885 | - | 1.18884 | 0.1714 |
| lipid | 0.44848 | 0.11274 | - | 1.19536 | 0.17329 |
| Canned tuna | 0.30022 | 0.03092 | - | 1.06588 | 0.17552 |
| Stir-fried pork | 0.37937 | 0.05699 | - | 1.12769 | 0.17839 |
| saturated fatty acids | 0.47801 | 0.12524 | - | 1.22718 | 0.18694 |
| Oils and fats | 0.34216 | 0.04512 | - | 1.16446 | 0.19652 |
| tap water | 1.66215 | 0.76139 | - | 3.85091 | 0.19983 |
| Whitebait | 0.27059 | 0.01708 | - | 1.1612 | 0.21305 |
| alcohol | 1.60671 | 0.74108 | - | 3.58888 | 0.21757 |
| grilled beef | 0.03304 | 0.00001 | - | 0.73703 | 0.22643 |
| theobromine | 1.57916 | 0.72661 | - | 3.47153 | 0.23214 |
| polyphenol | 1.57916 | 0.72661 | - | 3.47153 | 0.23214 |
| Chocolate | 1.57916 | 0.72661 | - | 3.47153 | 0.23214 |
| rice cake | 0.13816 | 0.00115 | - | 1.06736 | 0.23225 |
| Stewed pork | 0.19843 | 0.00589 | - | 1.1778 | 0.23249 |
| peach | 0.0114 | 0 | - | 1.04338 | 0.23423 |
| Sauce | 0.42264 | 0.06216 | - | 1.25253 | 0.24179 |
| Kamaboko (boiled fish paste) | 0.18237 | 0.00304 | - | 1.17512 | 0.24369 |
| Banana | 1.73747 | 0.79113 | - | 7.25507 | 0.24411 |
| garlic | 1.80802 | 0.81114 | - | 8.62911 | 0.24535 |
| 20:4 n-6arachidonic acid | 0.54509 | 0.16063 | - | 1.35027 | 0.25148 |
| Coffee (cans/plastic bottles) | 0.10232 | 0.00017 | - | 1.07189 | 0.25716 |
| Japanese tea (made from leaves) | 0.41321 | 0.04757 | - | 1.27826 | 0.266 |
| pacific saury | 0.56163 | 0.15894 | - | 1.3736 | 0.26832 |
| stir-fried chicken | 0.42699 | 0.0585 | - | 1.30981 | 0.27234 |
| Kiwi fruit | 1.54191 | 0.71567 | - | 4.51395 | 0.27547 |
| 20:0arachidic acid | 0.53037 | 0.13245 | - | 1.36759 | 0.27662 |
| manganese | 0.60126 | 0.21498 | - | 1.41402 | 0.2796 |
| miso soup | 1.51574 | 0.69138 | - | 3.39776 | 0.2806 |
| bread | 0.09371 | 0.00079 | - | 1.48293 | 0.28386 |
| organic acid | 1.54403 | 0.7103 | - | 4.5347 | 0.28439 |
| Coffee (instant) | 1.51846 | 0.69998 | - | 4.07673 | 0.28791 |
| fried chicken | 0.4116 | 0.05071 | - | 1.33825 | 0.28825 |
| Cod roe, sujiko(salted salmon roe), salmon roe | 0.13405 | 0.00069 | - | 1.21291 | 0.28849 |
| strawberry | 1.86687 | 0.81285 | - | 23.81533 | 0.29594 |
| apple | 0.56916 | 0.14351 | - | 1.41568 | 0.3063 |
| Watermelon | 0.10537 | 0.00016 | - | 1.05332 | 0.30718 |
| nori seaweed | 1.49432 | 0.67343 |  | 3.33878 | 0.3081 |
| vitamin B12 | 1.51341 | 0.69403 | - | 4.99987 | 0.30963 |
| 22:1docosenoic acid | 0.62116 | 0.21304 | - | 1.45577 | 0.31256 |
| vegetable juice | 1.47724 | 0.67579 | - | 3.72785 | 0.31293 |
| 22:5 n-6Docosapentaenoic acid | 0.6078 | 0.19098 | - | 1.45029 | 0.31571 |
| milk | 0.06546 | 0.00004 | - | 1.11616 | 0.31814 |
| Sesame | 1.46016 | 0.66376 | - | 3.18004 | 0.31851 |
| 14:0myristic acid | 0.60331 | 0.17559 | - | 1.45231 | 0.32244 |
| Pickled takuwan (pickled daikon radish) | 0.0174 | 0 | - | 1.16938 | 0.32259 |
| Yogurt | 1.44123 | 0.65991 | - | 3.35481 | 0.32364 |
| Pickled umeboshi (pickled plums) | 1.45378 | 0.66447 | - | 3.65748 | 0.3263 |
| Green onion/Wakegi (tree onion) | 0.04039 | 0.00001 | - | 1.55844 | 0.33003 |
| 21:5 n-3henicosapentaenoic acid | 0.62302 | 0.20104 | - | 1.47275 | 0.33036 |
| molybdenum | 1.46782 | 0.65923 | - | 3.30508 | 0.33157 |
| Chikuwa (tubular fish-paste) | 0.43159 | 0.04516 | - | 1.38804 | 0.33365 |
| Natto | 1.44283 | 0.65425 | - | 3.25023 | 0.3347 |
| n-6 polyunsaturated fatty acids | 0.57529 | 0.14979 | - | 1.45639 | 0.33604 |
| 15:0pentadecanoic acid | 0.61813 | 0.18692 | - | 1.47447 | 0.3365 |
| histidine | 0.62066 | 0.19146 | - | 1.48013 | 0.34 |
| β-cryptoxanthin | 0.23036 | 0.00548 | - | 1.30694 | 0.34126 |
| beef steak | 0.31886 | 0.00469 | - | 1.28568 | 0.34165 |
| polyunsaturated fatty acids | 0.58612 | 0.15738 | - | 1.46832 | 0.3432 |
| polyunsaturated fatty acids | 0.58632 | 0.15746 | - | 1.46863 | 0.34344 |
| 18:2n-6linoleic acid | 0.58503 | 0.15343 | - | 1.47124 | 0.34711 |
| 18:4 n-3Octadecatetraenoic acid | 0.6454 | 0.22418 | - | 1.50327 | 0.34793 |
| persimmon | 0.19371 | 0.00171 | - | 1.27805 | 0.34823 |
| 24:0lignoceric acid | 0.58365 | 0.14174 | - | 1.46845 | 0.3505 |
| 24:1Tetracosenoic acid | 0.63166 | 0.20104 | - | 1.49682 | 0.35084 |
| glycine | 0.62749 | 0.19429 | - | 1.49457 | 0.35121 |
| retinol | 0.49681 | 0.07219 | - | 1.43538 | 0.35377 |
| 22:5 n-3Docosapentaenoic acid | 0.63486 | 0.20387 | - | 1.50173 | 0.35407 |
| Boiled pork | 0.27474 | 0.00461 | - | 1.38724 | 0.35668 |
| Low ham | 0.50989 | 0.07449 | - | 1.4416 | 0.35779 |
| Rice crackers | 1.41047 | 0.63841 | - | 3.4849 | 0.35972 |
| Rice | 1.46283 | 0.64733 | - | 3.50683 | 0.36152 |
| grapes | 2.20253 | 0.86365 | - | 95.43215 | 0.36334 |
| fried pork | 0.28 | 0.00832 | - | 1.40135 | 0.36414 |
| pickled green leaves | 0.35077 | 0.01221 | - | 1.38121 | 0.36498 |
| 16:2Hexadecadienoic acid | 0.65469 | 0.21994 | - | 1.53014 | 0.37264 |
| Yam/Chinese yam | 0.60405 | 0.13538 | - | 1.50359 | 0.38182 |
| 16:4Hexadecatetraenoic acid | 0.65934 | 0.21863 | - | 1.54123 | 0.38282 |
| n-3 polyunsaturated fatty acids | 0.63415 | 0.18561 | - | 1.52889 | 0.38525 |
| mushrooms | 1.38668 | 0.62105 | - | 3.16192 | 0.3895 |
| Retinol activity equivalent | 0.59736 | 0.13556 | - | 1.51893 | 0.39446 |
| 22:0Behenic acid | 0.6328 | 0.16889 | - | 1.53315 | 0.39477 |
| Black tea (made from leaves) | 0.13267 | 0.00005 | - | 1.19041 | 0.39609 |
| carbonated drinks | 0.60071 | 0.10787 | - | 1.51111 | 0.39701 |
| egg | 1.41154 | 0.62624 | - | 3.24481 | 0.39724 |
| eggs | 1.41154 | 0.62624 | - | 3.24481 | 0.39724 |
| Sugar/sweeteners | 0.56932 | 0.06472 | - | 1.49118 | 0.39982 |
| 12:0Lauric acid | 0.61355 | 0.13626 | - | 1.52742 | 0.40122 |
| Snacks (potato chips) | 0.17651 | 0.00035 | - | 1.34118 | 0.40541 |
| vitamin D | 0.67997 | 0.235 | - | 1.57547 | 0.40801 |
| Enoki mushroom | 1.35866 | 0.60504 | - | 3.09502 | 0.40933 |
| 18:3n-6γ-linolenic acid | 0.66862 | 0.21976 | - | 1.56976 | 0.40941 |
| 18:3 n-3α-linolenic acid | 0.62825 | 0.15609 | - | 1.54978 | 0.41524 |
| caffeine | 1.35352 | 0.60067 | - | 3.19835 | 0.41666 |
| pork soup | 0.54203 | 0.0572 | - | 1.50229 | 0.42142 |
| Butter | 0.00154 | 0 | - | 0.53208 | 0.42749 |
| bird liver | 0.60077 | 0.09679 | - | 1.54238 | 0.43165 |
| mandarin orange | 0.34566 | 0.00883 | - | 1.39873 | 0.4329 |
| tannin | 1.3386 | 0.59054 | - | 3.13499 | 0.43347 |
| lycopene | 1.33934 | 0.59267 | - | 2.94137 | 0.43692 |
| coffee sugar | 0.59802 | 0.07369 | - | 1.5407 | 0.43769 |
| Vitamin B1 | 0.67665 | 0.20479 | - | 1.59671 | 0.43928 |
| daidzein | 1.34679 | 0.5965 | - | 2.9691 | 0.44214 |
| alanine | 0.69589 | 0.2346 | - | 1.61186 | 0.44237 |
| α-carotene | 1.34899 | 0.59743 | - | 2.95658 | 0.44598 |
| arginine | 0.70337 | 0.24677 | - | 1.62155 | 0.44718 |
| Genistein | 1.34188 | 0.59365 | - | 2.95996 | 0.44847 |
| Ketchup | 0.66686 | 0.16011 | - | 1.5946 | 0.45187 |
| 20:5 n-3Icosapentaenoic acid | 0.71086 | 0.25553 | - | 1.63251 | 0.45406 |
| methionine | 0.70465 | 0.23954 | - | 1.62636 | 0.45418 |
| noodle soup | 0.5286 | 0.05476 | - | 1.55266 | 0.46211 |
| pork liver | 0.56574 | 0.04839 | - | 1.5452 | 0.46402 |
| 22:6 n-3Docosahexaenoic acid | 0.72453 | 0.27224 | - | 1.65513 | 0.46905 |
| 16:3Hexadecatrienoic acid | 0.71407 | 0.24725 | - | 1.64447 | 0.46918 |
| boiled chicken | 0.65172 | 0.1185 | - | 1.59573 | 0.46924 |
| soba (buckwheat noodles) | 0.67723 | 0.16868 | - | 1.6141 | 0.46929 |
| deep-fried thin tofu | 0.63789 | 0.10534 | - | 1.58865 | 0.47067 |
| β-tocopherol | 0.70438 | 0.21737 | - | 1.63877 | 0.4754 |
| lysine | 0.71884 | 0.24825 | - | 1.65248 | 0.47649 |
| glutamic acid | 0.71147 | 0.2248 | - | 1.64947 | 0.48285 |
| Amino acid total | 0.72135 | 0.24597 | - | 1.65809 | 0.48302 |
| protein | 0.72218 | 0.24718 | - | 1.65907 | 0.48343 |
| Japanese pear | 0.23553 | 0.00089 | - | 1.40036 | 0.48418 |
| fruits | 1.29652 | 0.56046 | - | 3.06066 | 0.48637 |
| Protein by amino acid composition table | 0.72442 | 0.24794 | - | 1.66359 | 0.48774 |
| threonine | 0.72596 | 0.25204 | - | 1.66536 | 0.4878 |
| Pine | 0.44569 | 0.00852 | - | 1.51877 | 0.48887 |
| green asparagus | 1.29651 | 0.56653 | - | 2.8229 | 0.49775 |
| Pickled Chinese cabbage | 0.71689 | 0.20492 | - | 1.6601 | 0.49867 |
| biotin | 1.30592 | 0.5743 | - | 2.92562 | 0.50564 |
| proline | 0.71983 | 0.21636 | - | 1.6702 | 0.50666 |
| Total sulfur-containing amino acids | 0.73796 | 0.25895 | - | 1.68731 | 0.50717 |
| selenium | 0.73104 | 0.24125 | - | 1.68016 | 0.50741 |
| Wakame seaweed | 0.71188 | 0.19027 | - | 1.66663 | 0.51169 |
| Udon | 0.70703 | 0.19265 | - | 1.66576 | 0.51236 |
| Margarine | 0.68443 | 0.12096 | - | 1.65008 | 0.51817 |
| jam | 0.16314 | 0.00005 | - | 1.6766 | 0.51972 |
| green bell pepper | 0.5565 | 0.01488 | - | 1.56893 | 0.51973 |
| Commercial water | 0.62517 | 0.06561 | - | 1.62592 | 0.52171 |
| olive oil | 1.27532 | 0.55268 | - | 2.75375 | 0.52368 |
| Clams | 2.71176 | 0.83534 | - |  | 0.52708 |
| tryptophan | 0.75002 | 0.26545 | - | 1.71001 | 0.52785 |
| phenylalanine | 0.74924 | 0.2613 | - | 1.70964 | 0.52943 |
| Leucine | 0.74983 | 0.26172 | - | 1.71007 | 0.52952 |
| Serin | 0.75181 | 0.26536 | - | 1.71368 | 0.53176 |
| grains | 0.75773 | 0.28538 | - | 1.72475 | 0.53365 |
| Mayonnaise | 0.75299 | 0.26026 | - | 1.71646 | 0.53703 |
| 20:4 n-3Icosatetraenoic acid | 0.76345 | 0.28628 | - | 1.734 | 0.54259 |
| Total aromatic amino acids | 0.75852 | 0.26755 | - | 1.72641 | 0.54419 |
| Salt cod/salt salmon | 1.25725 | 0.53774 | - | 2.78638 | 0.54509 |
| isoleucine | 0.75991 | 0.26872 | - | 1.72853 | 0.54578 |
| Cake | 0.73566 | 0.2011 | - | 1.70285 | 0.54716 |
| 10:0Decanoic acid | 0.75516 | 0.24054 | - | 1.72273 | 0.55158 |
| Tyrosine | 0.76549 | 0.27166 | - | 1.73912 | 0.55574 |
| algae | 0.76461 | 0.25218 | - | 1.74326 | 0.56904 |
| Japanese sake | 1.23699 | 0.52037 | - | 2.61471 | 0.5692 |
| green bean | 0.74164 | 0.17953 | - | 1.7206 | 0.57167 |
| cholesterol | 0.78475 | 0.30837 | - | 1.7778 | 0.57562 |
| Beef stew | 0.709 | 0.12227 | - | 1.7081 | 0.57635 |
| Horse mackerel | 0.77253 | 0.25778 | - | 1.75279 | 0.57691 |
| Valin | 0.78097 | 0.28087 | - | 1.76786 | 0.58224 |
| Cystine | 0.78249 | 0.27876 | - | 1.77195 | 0.58797 |
| 8:0octanoic acid | 0.77633 | 0.24368 | - | 1.76042 | 0.59148 |
| zinc | 0.79073 | 0.29791 | - | 1.78763 | 0.59386 |
| sweet potato | 1.22438 | 0.51894 | - | 2.5995 | 0.59715 |
| 16:0isopalmitic acid | 0.78484 | 0.25733 | - | 1.7734 | 0.59998 |
| aspartic acid | 0.79424 | 0.29612 | - | 1.79431 | 0.60261 |
| soy milk | 1.22455 | 0.52448 | - | 2.59221 | 0.60337 |
| Wasabi | 1.22597 | 0.52762 | - | 2.69523 | 0.6052 |
| Niacin | 0.78992 | 0.27089 | - | 1.7848 | 0.60668 |
| Konjac | 0.78979 | 0.26431 | - | 1.78273 | 0.6071 |
| cucumber | 0.75164 | 0.13939 | - | 1.74433 | 0.60774 |
| γ-tocopherol | 0.78151 | 0.2443 | - | 1.78126 | 0.61228 |
| Energy (kcal) | 0.80618 | 0.31536 | - | 1.82157 | 0.6193 |
| magnesium | 1.22063 | 0.52893 | - | 2.71155 | 0.61934 |
| Energy (kJ) | 0.80652 | 0.31556 | - | 1.82228 | 0.61994 |
| Seasonings/Spices | 1.21898 | 0.52681 | - | 2.72864 | 0.62033 |
| green and yellow vegetables | 1.20877 | 0.50797 | - | 2.57384 | 0.62137 |
| ammonia | 0.79948 | 0.26227 | - | 1.80321 | 0.63084 |
| Burdock | 0.80189 | 0.2614 | - | 1.80252 | 0.63117 |
| Beer | 0.74777 | 0.09654 | - | 1.76218 | 0.63253 |
| carrot | 1.20837 | 0.51909 | - | 2.63192 | 0.63346 |
| Hijiki seaweed | 0.80184 | 0.26162 | - | 1.80601 | 0.63553 |
| 10:1Decenoic acid | 0.80633 | 0.27247 | - | 1.8112 | 0.63595 |
| Mixed oil | 0.73109 | 0.07862 | - | 1.75998 | 0.63724 |
| 4:0butyric acid | 0.80943 | 0.2713 | - | 1.81633 | 0.64255 |
| daikon (Japanese white radish) | 1.20227 | 0.5155 | - | 2.61834 | 0.64273 |
| Japanese sweets | 0.76491 | 0.13007 | - | 1.78096 | 0.64378 |
| vitamin K | 1.20047 | 0.51612 | - | 2.6316 | 0.64719 |
| 6:0hexanoic acid | 0.81456 | 0.27546 | - | 1.82557 | 0.65123 |
| salad dressing | 1.20015 | 0.52344 | - | 2.72681 | 0.65622 |
| coffee milk | 0.13687 | NA | - | 1.77375 | 0.66026 |
| potassium | 1.19189 | 0.51336 | - | 2.62879 | 0.66242 |
| tofu | 0.59177 | 0.00271 | - | 1.71191 | 0.66278 |
| 15:0 antpentadecanoic acid | 0.82176 | 0.28102 | - | 1.83839 | 0.66333 |
| Shimeji mushroom | 1.1822 | 0.48749 | - | 2.5125 | 0.66339 |
| Chive | 0.69954 | NA | - | 1.7529 | 0.66516 |
| Shrimp | 1.17983 | 0.48817 | - | 2.51965 | 0.66865 |
| Shungiku (garland chrysanthemum) | 0.67546 | NA |  | 1.7427 | 0.67108 |
| 17:0 antheptadecanoic acid | 0.82837 | 0.28645 | - | 1.85058 | 0.67471 |
| pickled eggplant | 0.81741 | 0.22745 | - | 1.83112 | 0.67536 |
| vitamin B2 | 0.83327 | 0.30711 | - | 1.86562 | 0.6785 |
| Pickled cucumber | 1.17071 | 0.4734 | - | 2.4936 | 0.68001 |
| beans | 1.1661 | 0.49504 | - | 2.55905 | 0.70038 |
| tomato | 1.15207 | 0.44423 | - | 2.48601 | 0.70797 |
| gross weight | 0.85189 | 0.33843 | - | 1.91634 | 0.70819 |
| moisture | 0.85194 | 0.33643 | - | 1.9154 | 0.70901 |
| potato | 0.8494 | 0.31239 | - | 1.89379 | 0.70927 |
| acetic acid | 0.85325 | 0.33097 | - | 1.91231 | 0.71327 |
| sea ​​bream | 0.84621 | 0.26405 | - | 1.87645 | 0.71701 |
| 13:0tridecanoic acid | 1.15148 | 0.47007 | - | 2.45537 | 0.71769 |
| 7:0heptanoic acid | 1.14947 | 0.46859 | - | 2.45096 | 0.72115 |
| α-tocopherol | 0.85682 | 0.31672 | - | 1.91218 | 0.72498 |
| Fruit drinks (not 100%) | 1.14322 | 0.44927 | - | 2.39215 | 0.72795 |
| vegetables | 1.14363 | 0.46841 | - | 2.44799 | 0.73264 |
| Yakult | 1.13985 | 0.45789 | - | 2.40686 | 0.73698 |
| Confectionery | 1.13902 | 0.45917 | - | 2.42057 | 0.73891 |
| nitrate ion | 0.8491 | 0.19523 | - | 1.8953 | 0.74345 |
| Milks | 1.13759 | 0.46544 | - | 2.4402 | 0.74351 |
| alcoholic drinks | 1.13316 | 0.45102 | - | 2.47694 | 0.74763 |
| copper | 1.14006 | 0.49378 | - | 2.57866 | 0.74961 |
| Pickled turnips | 1.12925 | 0.46035 | - | 2.40627 | 0.75847 |
| water soluble dietary fiber | 1.13244 | 0.48625 | - | 2.52568 | 0.76062 |
| cabbage | 1.12467 | 0.47575 | - | 2.47194 | 0.77159 |
| Somen/Hiyamugi | 0.87887 | 0.28938 | - | 1.93368 | 0.77285 |
| Japanese mustard | 1.10902 | 0.44433 | - | 2.40067 | 0.79385 |
| Octopus | 1.10188 | 0.40812 | - | 2.35877 | 0.8027 |
| Bisket | 1.10114 | 0.45137 | - | 2.37848 | 0.81049 |
| bean sprouts | 0.90137 | 0.30885 | - | 1.98412 | 0.81401 |
| Lettuce | 1.09962 | 0.45839 | - | 2.39374 | 0.81463 |
| calcium | 1.098 | 0.44443 | - | 2.36553 | 0.81536 |
| deep-fried thick tofu | 0.90273 | 0.30304 | - | 1.98189 | 0.81692 |
| soup | 1.09571 | 0.43148 | - | 2.36307 | 0.81776 |
| Peanuts | 0.90291 | 0.28714 | - | 1.96635 | 0.81782 |
| citrus | 0.89921 | 0.20611 | - | 1.95855 | 0.81909 |
| cooking salt | 0.90461 | 0.30984 | - | 1.99147 | 0.82018 |
| Japanese mustard spinach | 1.09155 | 0.39288 | - | 2.34545 | 0.82175 |
| spinach | 0.90884 | 0.35022 | - | 2.01024 | 0.82268 |
| Tomato juice | 0.90343 | 0.22029 | - | 1.96787 | 0.82551 |
| phosphorus | 0.91283 | 0.35199 | - | 2.0192 | 0.83067 |
| chromium | 0.9172 | 0.3484 | - | 2.02417 | 0.83987 |
| Broccoli | 1.08189 | 0.3985 | - | 2.35205 | 0.84121 |
| coffee (made from beans) | 0.91642 | 0.26044 | - | 1.99698 | 0.84598 |
| Eggplant | 0.91497 | 0.20386 | - | 1.99829 | 0.8473 |
| Total amount of dietary fiber | 1.08135 | 0.45677 | - | 2.39475 | 0.84848 |
| carbohydrates | 0.92312 | 0.36832 | - | 2.05633 | 0.84998 |
| Seeds and fruits | 0.92203 | 0.29994 | - | 1.99931 | 0.85267 |
| ice cream | 0.92214 | 0.29791 | - | 2.00584 | 0.85336 |
| ash | 1.06916 | 0.44989 | - | 2.37449 | 0.87038 |
| seafood | 1.06323 | 0.43695 | - | 2.32885 | 0.88051 |
| insoluble dietary fiber | 1.06213 | 0.44375 | - | 2.34209 | 0.88305 |
| Available carbohydrates (monosaccharide equivalents) | 0.94262 | 0.37289 | - | 2.09075 | 0.88864 |
| vitamin B6 | 0.94401 | 0.37218 | - | 2.08614 | 0.89133 |
| β-carotene equivalent | 0.94523 | 0.33763 | - | 2.05746 | 0.89514 |
| spaghetti macaroni | 0.94799 | 0.34216 | - | 2.07251 | 0.90042 |
| vitamin C | 1.04887 | 0.41006 | - | 2.25693 | 0.90655 |
| Pickles | 0.95613 | 0.36547 | - | 2.08707 | 0.91524 |
| onion | 0.95656 | 0.30764 | - | 2.05496 | 0.9174 |
| squid | 1.04219 | 0.39278 | - | 2.23321 | 0.9189 |
| water | 1.04035 | 0.39515 | - | 2.2576 | 0.9224 |
| δ-tocopherol | 1.04031 | 0.42084 | - | 2.27571 | 0.92317 |
| Salt equivalent amount | 0.96614 | 0.39402 | - | 2.15442 | 0.93432 |
| iron | 1.03346 | 0.43874 | - | 2.32251 | 0.93653 |
| Potatoes | 0.96763 | 0.37991 | - | 2.1219 | 0.93741 |
| sodium | 0.9705 | 0.39651 | - | 2.16396 | 0.94284 |
| folic acid | 0.97097 | 0.38892 | - | 2.14019 | 0.94381 |
| Cheese | 1.02455 | 0.41323 | - | 2.23691 | 0.95304 |
| 22:2docosadienoic acid | 1.02355 | 0.40906 | - | 2.22756 | 0.95492 |
| Salmon | 1.02355 | 0.40906 | - | 2.22756 | 0.95492 |
| Japanese leek | 1.0197 | 0.30104 | - | 2.20018 | 0.96174 |
| iodine | 0.98389 | 0.38931 | - | 2.1528 | 0.96892 |
| pumpkin | 0.98431 | 0.32243 | - | 2.11525 | 0.96995 |
| Dried fish | 0.9847 | 0.3907 | - | 2.15342 | 0.97047 |
| Satsuma-age (fried fish paste) | 0.98489 | 0.35307 | - | 2.11417 | 0.97096 |
| boiled tofu | 0.98923 | 0.3954 | - | 2.17188 | 0.97923 |
| Yellowtail | 1.00899 | 0.39429 | - | 2.18187 | 0.98273 |
| β-carotene | 1.00415 | 0.36813 | - | 2.1725 | 0.99202 |
| pantothenic acid | 0.99678 | 0.40887 | - | 2.20567 | 0.9938 |
| whiskey | 0.00267 | NA | - | 2.4962E+50 | 0.99393 |
| Oolong tea (cans/plastic bottles) | 0 | NA | - | 2.80318E+74 | 0.99434 |
| Eel | 0 | NA | - | 3.62377E+74 | 0.99439 |
| Melon | 0 | NA | - | 1.2309E+214 | 0.99457 |
| wine | 0.06445 | NA | - | 2.13569E+36 | 0.99484 |
| black tea sugar | 0.06445 | NA | - | 2.13569E+36 | 0.99484 |
| soybean oil | 0.06445 | NA | - | 2.13569E+36 | 0.99484 |
| Black tea (cans/plastic bottles) | 0 | NA | - | 1.943E+99 | 0.99491 |
| safflower oil | 0 | NA | - | 6.4263E+201 | 0.99494 |
| Rapeseed oil/canola oil | 0 | NA | - | Infinite | 0.99511 |
| Koya tofu (freeze-dried tofu) | 0 | NA | - | 3.8797E+301 | 0.99539 |
| low fat milk | 0 | NA | - | Infinite | 0.99541 |
| Oolong tea (made from leaves) | 0 | NA | - | 2.7339E+138 | 0.99557 |
| others | 0 | NA | - | 4.759E+184 | 0.9957 |
| Orange juice | 0.00083 | NA | - | 3.90417E+87 | 0.99575 |
| Apple juice | 0.00083 | NA | - | 3.90417E+87 | 0.99575 |
| Other vegetables | 0.99781 | 0.38403 | - | 2.16492 | 0.99578 |
| tea milk | 0 | NA | - | Infinite | 0.99589 |
| Tara | 1 | 0.38799 | - | 2.16583 | 1 |
| cooking oil | NA | NA | - | NA | NA |
| 15:1pentadecenoic acid | NA | NA | - | NA | NA |
| 17:2Heptadecadienoic acid | NA | NA | - | NA | NA |
| 18:2Octadecadienoic acid | NA | NA | - | NA | NA |
| 18:3Octadecatrienoic acid | NA | NA | - | NA | NA |
| 100% grapefruit juice | NA | NA | - | NA | NA |
| corn oil | NA | NA | - | NA | NA |

NA, not available.

**Table S3.** Odds ratios for 330 food or nutrition items (adjusted for energy [per kilocalorie] and converted into z-scores) in the univariate logistic regression analyses for the risk of non-objective response, ranked according to P value.

| **Food or nutrition items** | **Odds ratio** | **95% CI (low)** |  | **95% CI (high)** | **P value** |
| --- | --- | --- | --- | --- | --- |
| acetic acid | 0.34676 | 0.12589 | - | 0.77843 | 0.01902 |
| Bacon | 0.25841 | 0.06548 | - | 0.6727 | 0.02045 |
| 20:2 n-6Icosadienoic acid | 0.34289 | 0.11535 | - | 0.79181 | 0.02522 |
| Mayonnaise | 0.29321 | 0.07788 | - | 0.73089 | 0.0277 |
| 16:0palmitic acid | 0.3761 | 0.1378 | - | 0.84563 | 0.03009 |
| Total amount of fatty acids | 0.37754 | 0.13864 | - | 0.85155 | 0.03125 |
| Triacylglycerol equivalent | 0.3778 | 0.13872 | - | 0.85212 | 0.03136 |
| Lipid | 0.37854 | 0.13939 | - | 0.85372 | 0.0314 |
| 18:1Total | 0.39028 | 0.1421 | - | 0.87671 | 0.03838 |
| Sauce | 0.37226 | 0.12054 | - | 0.84791 | 0.04012 |
| stir-fried chicken | 0.41917 | 0.16462 | - | 0.90645 | 0.04021 |
| Boiled pork | 0.28893 | 0.07146 | - | 0.78303 | 0.04148 |
| monounsaturated fatty acids | 0.40282 | 0.14928 | - | 0.89815 | 0.04239 |
| saturated fatty acids | 0.40539 | 0.15009 | - | 0.90368 | 0.043 |
| 18:0stearic acid | 0.40897 | 0.15329 | - | 0.90994 | 0.04412 |
| 20:4 n-6arachidonic acid | 0.38368 | 0.12986 | - | 0.9023 | 0.04736 |
| 22:4 n-6docosatetraenoic acid | 0.3664 | 0.11204 | - | 0.86253 | 0.0476 |
| pork soup | 0.34861 | 0.09439 | - | 0.84067 | 0.05263 |
| 20:0arachidic acid | 0.4299 | 0.16107 | - | 0.95012 | 0.05442 |
| Hijiki seaweed | 0.35896 | 0.10161 | - | 0.89198 | 0.06505 |
| Wiener sausage | 0.40259 | 0.12795 | - | 0.93194 | 0.06753 |
| Margarine | 0.16149 | 0.01527 | - | 0.71067 | 0.06959 |
| 16:1palmitoleic acid | 0.43957 | 0.15942 | - | 0.9768 | 0.06978 |
| Green onion/Wakegi (tree onion) | 0.09125 | 0.00458 | - | 0.77964 | 0.07237 |
| Meat | 0.47219 | 0.18738 | - | 1.01447 | 0.07296 |
| 20:3 n-6Icosatrienoic acid | 0.4745 | 0.19074 | - | 1.02344 | 0.07445 |
| 18:1n-9oleic acid | 0.33593 | 0.08469 | - | 0.9007 | 0.07837 |
| Oils and fats | 0.34652 | 0.08662 | - | 0.90592 | 0.08104 |
| Seasonings/Spices | 0.49097 | 0.1988 | - | 1.05783 | 0.08638 |
| 18:1n-7cis-vaccenic acid | 0.37791 | 0.10523 | - | 0.94214 | 0.08641 |
| n-6 polyunsaturated fatty acids | 0.48983 | 0.1946 | - | 1.06015 | 0.08959 |
| salad dressing | 0.5083 | 0.2142 | - | 1.0769 | 0.09165 |
| soba (buckwheat noodles) | 3.15693 | 1.08778 | - | 16.68476 | 0.0935 |
| Ketchup | 0.40229 | 0.10013 | - | 0.95199 | 0.09552 |
| 18:2n-6linoleic acid | 0.49904 | 0.20013 | - | 1.07648 | 0.09574 |
| 18:3 n-3α-linolenic acid | 0.50615 | 0.20197 | - | 1.09017 | 0.10288 |
| polyunsaturated fatty acids | 0.50826 | 0.20301 | - | 1.09858 | 0.10594 |
| polyunsaturated fatty acids | 0.50877 | 0.2033 | - | 1.09951 | 0.10633 |
| chromium | 2.30784 | 0.95226 | - | 7.55627 | 0.10634 |
| daikon (Japanese white radish) | 2.19579 | 0.9373 | - | 6.71754 | 0.10745 |
| 24:0lignoceric acid | 0.52713 | 0.21201 | - | 1.11453 | 0.11414 |
| 17:0heptadecanoic acid | 0.52782 | 0.21935 | - | 1.12233 | 0.11463 |
| Taro | 2.44525 | 0.93095 | - | 9.99408 | 0.13115 |
| iodine | 0.53183 | 0.20385 | - | 1.14201 | 0.13919 |
| Coffee (cans/plastic bottles) | 0.5506 | 0.21402 | - | 1.15363 | 0.13942 |
| 14:0myristic acid | 0.53161 | 0.19827 | - | 1.1496 | 0.14622 |
| nori seaweed | 2.54701 | 0.92691 | - | 13.13099 | 0.15267 |
| boiled tofu | 3.79748 | 0.92197 | - | 32.32776 | 0.15312 |
| α-carotene | 2.42552 | 0.90975 | - | 10.59464 | 0.15399 |
| carbonated drinks | 5.95606 | 1.27113 | - | 517.78287 | 0.15527 |
| Sugar/sweeteners | 0.54762 | 0.19676 | - | 1.16264 | 0.15592 |
| Japanese tea (made from leaves) | 0.58864 | 0.2573 | - | 1.22058 | 0.16391 |
| Oolong tea (cans/plastic bottles) | 0.58335 | 0.24312 | - | 1.21118 | 0.164 |
| jam | 0.02346 | 0.00002 | - | 0.79143 | 0.16537 |
| 17:1Heptadecenoic acid | 0.56242 | 0.21946 | - | 1.19859 | 0.16778 |
| Rapeseed oil/canola oil | 0.55924 | 0.19004 | - | 1.18357 | 0.17051 |
| 12:0Lauric acid | 0.54874 | 0.19409 | - | 1.18179 | 0.1711 |
| vitamin B6 | 1.86616 | 0.83063 | - | 5.28965 | 0.17139 |
| carrot | 2.03605 | 0.84354 | - | 6.93751 | 0.17726 |
| 22:2docosadienoic acid | 1.85273 | 0.82414 | - | 5.29049 | 0.17741 |
| Salmon | 1.85273 | 0.82414 | - | 5.29049 | 0.17741 |
| 20:1Icosenoic acid | 0.58991 | 0.25614 | - | 1.25262 | 0.18302 |
| magnesium | 1.8346 | 0.81575 | - | 5.15871 | 0.18465 |
| tofu | 11.69866 | 0.95357 | - | 1103.07649 | 0.18845 |
| cholesterol | 0.57431 | 0.22539 | - | 1.25323 | 0.19276 |
| coffee sugar | 0.58596 | 0.21469 | - | 1.23314 | 0.19423 |
| 15:0pentadecanoic acid | 0.59025 | 0.2351 | - | 1.25218 | 0.19779 |
| Somen/Hiyamugi | 1.9024 | 0.81582 | - | 6.35717 | 0.19968 |
| fried pork | 0.59834 | 0.2378 | - | 1.26045 | 0.20448 |
| 14:1myristoleic acid | 0.61077 | 0.26248 | - | 1.28022 | 0.20451 |
| Available carbohydrates (monosaccharide equivalents) | 1.73118 | 0.78325 | - | 4.55713 | 0.21226 |
| Butter | 0.16483 | 0.0059 | - | 1.03421 | 0.21507 |
| 22:0Behenic acid | 0.62106 | 0.26897 | - | 1.29991 | 0.21644 |
| theobromine | 1.91059 | 0.80225 | - | 6.69818 | 0.21798 |
| polyphenol | 1.91059 | 0.80225 | - | 6.69818 | 0.21798 |
| Chocolate | 1.91059 | 0.80225 | - | 6.69818 | 0.21798 |
| Stewed pork | 0.62085 | 0.25135 | - | 1.2971 | 0.22387 |
| 18:3n-6γ-linolenic acid | 1.77249 | 0.77871 | - | 5.28991 | 0.22932 |
| rice cake | 0.63704 | 0.2802 | - | 1.3215 | 0.23052 |
| Japanese leek | 0.5623 | 0.14656 | - | 1.23402 | 0.23514 |
| alcohol | 1.71446 | 0.76794 | - | 5.01799 | 0.23775 |
| Bisket | 1.79978 | 0.77843 | - | 6.00709 | 0.23783 |
| Stir-fried pork | 0.61645 | 0.23642 | - | 1.30549 | 0.24035 |
| alcoholic drinks | 2.32025 | 0.81803 | - | 13.70582 | 0.24254 |
| fruits | 2.43964 | 0.82973 | - | 16.70667 | 0.24375 |
| copper | 1.70648 | 0.76136 | - | 4.81084 | 0.245 |
| 10:0Decanoic acid | 0.62849 | 0.25177 | - | 1.32472 | 0.2474 |
| Cod roe, sujiko(salted salmon roe), salmon roe | 0.63193 | 0.24951 | - | 1.32696 | 0.24999 |
| beef steak | 3.37466 | 0.90865 | - | 96.82118 | 0.25282 |
| Canned tuna | 0.64869 | 0.28174 | - | 1.35244 | 0.2544 |
| Low ham | 0.64688 | 0.27469 | - | 1.34994 | 0.25649 |
| deep-fried thick tofu | 1.88752 | 0.76948 | - | 7.27108 | 0.26113 |
| squid | 1.74946 | 0.75987 | - | 6.10278 | 0.26296 |
| organic acid | 2.1274 | 0.79153 | - | 11.61974 | 0.26374 |
| 16:0isopalmitic acid | 0.64207 | 0.26075 | - | 1.3505 | 0.26385 |
| n-3 polyunsaturated fatty acids | 0.64644 | 0.27847 | - | 1.37214 | 0.27066 |
| 4:0butyric acid | 0.64974 | 0.26443 | - | 1.36625 | 0.27483 |
| Total amount of dietary fiber | 1.64691 | 0.73932 | - | 4.64811 | 0.27486 |
| folic acid | 1.61463 | 0.73404 | - | 4.32018 | 0.27716 |
| γ-tocopherol | 0.65965 | 0.29216 | - | 1.38916 | 0.28151 |
| pickled green leaves | 1.89848 | 0.76851 | - | 9.98201 | 0.28258 |
| insoluble dietary fiber | 1.61455 | 0.73135 | - | 4.42525 | 0.28335 |
| water soluble dietary fiber | 1.64192 | 0.73467 | - | 4.73475 | 0.28355 |
| 8:0octanoic acid | 0.65712 | 0.26984 | - | 1.38112 | 0.2845 |
| carbohydrates | 1.55077 | 0.72191 | - | 3.76502 | 0.28589 |
| 15:0 antpentadecanoic acid | 0.66468 | 0.28033 | - | 1.39493 | 0.2917 |
| bird liver | 3.14604 | 0.81929 | - | 63.83259 | 0.29234 |
| 10:1Decenoic acid | 0.66507 | 0.27995 | - | 1.39623 | 0.29295 |
| Eel | 0.67626 | 0.29432 | - | 1.40593 | 0.29316 |
| pickled eggplant | 1.75237 | 0.73965 | - | 7.34288 | 0.30449 |
| Pickles | 1.61863 | 0.72236 | - | 5.09759 | 0.30714 |
| β-cryptoxanthin | 2.10401 | 0.7655 | - | 16.21494 | 0.32144 |
| 17:0 antheptadecanoic acid | 0.68414 | 0.29318 | - | 1.43638 | 0.32185 |
| 6:0hexanoic acid | 0.68548 | 0.2893 | - | 1.44093 | 0.32678 |
| Luxury drinks | 0.69322 | 0.32042 | - | 1.45049 | 0.32719 |
| olive oil | 1.59306 | 0.70982 | - | 5.27737 | 0.33043 |
| Snacks (potato chips) | 0.69795 | 0.30338 | - | 1.4566 | 0.33193 |
| Chive | 0.56554 | 0.07302 | - | 1.3012 | 0.3406 |
| Dried fish | 1.52057 | 0.69467 | - | 4.33012 | 0.34524 |
| Chinese cabbage | 1.51624 | 0.69286 | - | 4.34358 | 0.34893 |
| vitamin C | 1.61949 | 0.70224 | - | 5.74704 | 0.35287 |
| pumpkin | 1.55593 | 0.69484 | - | 5.07944 | 0.35608 |
| Yellowtail | 0.68826 | 0.24089 | - | 1.4641 | 0.3574 |
| Broccoli | 1.83476 | 0.72126 | - | 12.96927 | 0.36453 |
| Pickled cucumber | 1.94687 | 0.73461 | - | 19.48535 | 0.36994 |
| Stir-fried beef | 0.70347 | 0.25926 | - | 1.49379 | 0.3735 |
| coffee (made from beans) | 0.71811 | 0.30262 | - | 1.5129 | 0.37912 |
| apple | 0.72337 | 0.33615 | - | 1.51752 | 0.38392 |
| ice cream | 1.55142 | 0.68481 | - | 6.03654 | 0.38524 |
| mandarin orange | 1.74845 | 0.70591 | - | 11.58828 | 0.38617 |
| Beer | 1.97503 | 0.72594 | - | 26.55682 | 0.3874 |
| gross weight | 1.4034 | 0.66247 | - | 3.23741 | 0.38898 |
| green asparagus | 1.62657 | 0.68696 | - | 6.93624 | 0.39277 |
| Wasabi | 1.41663 | 0.66107 | - | 3.56232 | 0.40211 |
| onion | 1.57088 | 0.67863 | - | 6.82947 | 0.40683 |
| β-carotene equivalent | 1.47694 | 0.66812 | - | 4.83659 | 0.40687 |
| Salt cod/salt salmon | 1.4505 | 0.66312 | - | 4.38016 | 0.41181 |
| pantothenic acid | 1.3963 | 0.65467 | - | 3.37771 | 0.41237 |
| vitamin B12 | 2.26932 | 0.70787 | - | 23.92738 | 0.415 |
| moisture | 1.37458 | 0.64938 | - | 3.15601 | 0.41735 |
| Fruit drinks (not 100%) | 0.72835 | 0.24912 | - | 1.56219 | 0.41888 |
| Other vegetables | 1.44248 | 0.6562 | - | 4.20767 | 0.42508 |
| seafood | 1.3622 | 0.6427 | - | 3.13964 | 0.43458 |
| calcium | 1.38197 | 0.64596 | - | 3.46944 | 0.43597 |
| Banana | 1.68956 | 0.67729 | - | 11.99458 | 0.43864 |
| Shungiku (garland chrysanthemum) | 0.41064 | 0.0136 | - | 1.203 | 0.43899 |
| Tomato juice | 1.5383 | 0.6634 | - | 7.73856 | 0.44003 |
| vegetables | 1.43059 | 0.65208 | - | 4.67159 | 0.44033 |
| Pickled takuwan (pickled daikon radish) | 1.71864 | 0.68731 | - | 25.82751 | 0.44233 |
| Black tea (cans/plastic bottles) | 0.75557 | 0.33702 | - | 1.60136 | 0.4445 |
| coffee milk | 0.06147 | 0 | - | 0.94535 | 0.4518 |
| Whitebait | 1.42309 | 0.64419 | - | 4.55589 | 0.45777 |
| Tara | 0.76026 | 0.35137 | - | 1.60599 | 0.45926 |
| Commercial water | 0.76251 | 0.3471 | - | 1.61616 | 0.46026 |
| tap water | 1.36225 | 0.63747 | - | 3.57449 | 0.46038 |
| grains | 1.33937 | 0.6314 | - | 3.16231 | 0.46407 |
| 22:1docosenoic acid | 1.33715 | 0.63053 | - | 3.16937 | 0.46789 |
| boiled chicken | 0.7654 | 0.35534 | - | 1.61808 | 0.46887 |
| Enoki mushroom | 1.62687 | 0.66237 | - | 15.84388 | 0.47355 |
| pacific saury | 1.33381 | 0.62786 | - | 3.27848 | 0.47895 |
| Yogurt | 1.38547 | 0.63362 | - | 4.83679 | 0.48585 |
| α-tocopherol | 0.76995 | 0.35564 | - | 1.62552 | 0.48686 |
| Wakame seaweed | 1.35806 | 0.62969 | - | 4.01154 | 0.48733 |
| noodle soup | 1.37823 | 0.62993 | - | 4.51991 | 0.49187 |
| Japanese tea (cans/plastic bottles) | 1.53249 | 0.64318 | - | 10.3136 | 0.49893 |
| Shrimp | 1.36457 | 0.62527 | - | 4.35832 | 0.50196 |
| pork liver | 0.78452 | 0.35892 | - | 1.67489 | 0.50702 |
| garlic | 1.73896 | 0.65963 | - | 26.40266 | 0.50893 |
| grilled beef | 0.78507 | 0.33669 | - | 1.69316 | 0.5123 |
| glycine | 0.77635 | 0.34612 | - | 1.63878 | 0.51314 |
| Mixed oil | 0.78664 | 0.34776 | - | 1.68649 | 0.51583 |
| Shiitake mushroom | 1.33354 | 0.61629 | - | 3.81716 | 0.51772 |
| cabbage | 1.30831 | 0.6145 | - | 3.37556 | 0.51789 |
| Cake | 1.2936 | 0.61182 | - | 3.06206 | 0.51887 |
| phosphorus | 1.2852 | 0.60805 | - | 2.93205 | 0.52113 |
| egg | 0.785 | 0.36531 | - | 1.65621 | 0.52151 |
| eggs | 0.785 | 0.36531 | - | 1.65621 | 0.52151 |
| Koya tofu (freeze-dried tofu) | 1.37177 | 0.62061 | - | 5.60651 | 0.52322 |
| molybdenum | 1.2978 | 0.60985 | - | 3.38097 | 0.53106 |
| iron | 1.2749 | 0.60337 | - | 2.91577 | 0.53455 |
| green and yellow vegetables | 1.32379 | 0.61097 | - | 4.2918 | 0.53873 |
| daidzein | 1.28794 | 0.60623 | - | 3.25659 | 0.5388 |
| Confectionery | 1.32386 | 0.61 | - | 4.15477 | 0.53907 |
| beans | 1.26995 | 0.60209 | - | 2.96846 | 0.54547 |
| β-carotene | 1.28814 | 0.60496 | - | 3.60537 | 0.55043 |
| Rice crackers | 1.39041 | 0.61379 | - | 8.12633 | 0.55059 |
| 16:4Hexadecatetraenoic acid | 1.25704 | 0.5962 | - | 2.82926 | 0.55572 |
| 21:5 n-3henicosapentaenoic acid | 1.25832 | 0.59653 | - | 2.86344 | 0.55615 |
| lycopene | 1.32461 | 0.60512 | - | 4.83214 | 0.55716 |
| Genistein | 1.26877 | 0.59872 | - | 3.14753 | 0.55889 |
| grapes | 2.94264 | 0.63261 | - | 538.37018 | 0.56285 |
| proline | 1.25102 | 0.59163 | - | 2.91478 | 0.57073 |
| Japanese sake | 1.30439 | 0.60253 | - | 5.69859 | 0.57086 |
| Beef stew | 1.28297 | 0.59794 | - | 3.91938 | 0.57209 |
| 7:0heptanoic acid | 1.26082 | 0.59539 | - | 3.24353 | 0.57314 |
| mushrooms | 1.33682 | 0.60102 | - | 5.9774 | 0.57354 |
| Eggplant | 1.4377 | 0.61119 | - | 15.55393 | 0.57429 |
| 18:4 n-3Octadecatetraenoic acid | 1.24341 | 0.5896 | - | 2.82167 | 0.57598 |
| 13:0tridecanoic acid | 1.25762 | 0.5941 | - | 3.22942 | 0.57688 |
| potassium | 1.24931 | 0.59212 | - | 2.99892 | 0.57695 |
| Vitamin B1 | 0.81159 | 0.37402 | - | 1.72111 | 0.57929 |
| strawberry | 1.55603 | 0.6255 | - | 46.33437 | 0.57953 |
| Pickled turnips | 1.26179 | 0.59437 | - | 3.57202 | 0.58229 |
| citrus | 0.81977 | 0.35919 | - | 1.80386 | 0.58521 |
| 16:3Hexadecatrienoic acid | 1.23707 | 0.58684 | - | 2.81503 | 0.58538 |
| Shochu | 1.25368 | 0.59293 | - | 3.45718 | 0.58712 |
| vitamin D | 1.22754 | 0.58146 | - | 2.704 | 0.59272 |
| Kiwi fruit | 4.57619 | 0.68999 | - | 58324.70181 | 0.59668 |
| Chikuwa (tubular fish-paste) | 0.8279 | 0.38434 | - | 1.79327 | 0.60594 |
| 16:2Hexadecadienoic acid | 1.21839 | 0.57817 | - | 2.71019 | 0.60841 |
| vegetable juice | 1.27109 | 0.58362 | - | 5.00868 | 0.61387 |
| spinach | 1.22084 | 0.5793 | - | 3.03421 | 0.62072 |
| Lettuce | 1.21599 | 0.57767 | - | 2.94054 | 0.62394 |
| 20:4 n-3Icosatetraenoic acid | 1.20271 | 0.5698 | - | 2.62174 | 0.62832 |
| sea ​​bream | 1.21073 | 0.57527 | - | 2.9529 | 0.63264 |
| glutamic acid | 1.20172 | 0.56934 | - | 2.66912 | 0.63274 |
| Clams | 1.77145 | 0.62624 | - | NA | 0.63376 |
| ash | 1.20285 | 0.57147 | - | 2.75737 | 0.6357 |
| Kamaboko (boiled fish paste) | 0.84298 | 0.38618 | - | 1.85962 | 0.63879 |
| biotin | 1.19785 | 0.56882 | - | 2.78256 | 0.64552 |
| grilled chicken | 0.84775 | 0.39655 | - | 1.84433 | 0.65288 |
| Potatoes | 1.20077 | 0.5672 | - | 3.16172 | 0.65711 |
| Milks | 1.19026 | 0.5663 | - | 2.8134 | 0.65886 |
| Retinol activity equivalent | 1.18983 | 0.56515 | - | 3.02085 | 0.66713 |
| 22:5 n-3Docosapentaenoic acid | 0.84914 | 0.39123 | - | 1.79376 | 0.66732 |
| ammonia | 1.17354 | 0.5561 | - | 2.63287 | 0.67831 |
| Peanuts | 0.86032 | 0.40669 | - | 1.88308 | 0.68166 |
| sweet potato | 1.18267 | 0.56052 | - | 3.16051 | 0.68275 |
| algae | 1.17736 | 0.56093 | - | 2.98092 | 0.68436 |
| 20:5 n-3Icosapentaenoic acid | 1.16481 | 0.55195 | - | 2.53293 | 0.68883 |
| 22:5 n-6Docosapentaenoic acid | 1.16413 | 0.55235 | - | 2.5549 | 0.69106 |
| Seeds and fruits | 0.86488 | 0.4087 | - | 1.89816 | 0.69232 |
| Yam/Chinese yam | 0.86979 | 0.4101 | - | 1.90334 | 0.70492 |
| Konjac | 1.16573 | 0.55048 | - | 3.13869 | 0.7091 |
| alanine | 0.86916 | 0.39563 | - | 1.8317 | 0.71363 |
| Octopus | 1.16216 | 0.55059 | - | 3.25818 | 0.71553 |
| Burdock | 0.87479 | 0.41895 | - | 1.88115 | 0.71923 |
| 24:1Tetracosenoic acid | 0.87367 | 0.4024 | - | 1.84343 | 0.72269 |
| fried chicken | 0.87805 | 0.41655 | - | 1.91648 | 0.72495 |
| spaghetti macaroni | 1.1488 | 0.54626 | - | 2.88725 | 0.72841 |
| Horse mackerel | 1.14401 | 0.54564 | - | 2.64731 | 0.72947 |
| Yakult | 0.88151 | 0.41309 | - | 1.978 | 0.73019 |
| Cystine | 1.13884 | 0.53649 | - | 2.44683 | 0.73093 |
| Pickled Chinese cabbage | 1.14198 | 0.54648 | - | 2.68159 | 0.73374 |
| Japanese pear | 1.15022 | 0.54596 | - | 3.56626 | 0.7359 |
| cucumber | 1.15471 | 0.54049 | - | 3.85692 | 0.73616 |
| lysine | 0.88218 | 0.40358 | - | 1.8595 | 0.74233 |
| tannin | 0.89282 | 0.39388 | - | 2.10729 | 0.75509 |
| selenium | 1.12533 | 0.53056 | - | 2.43076 | 0.75534 |
| soup | 1.12963 | 0.53984 | - | 2.72435 | 0.75615 |
| Japanese sweets | 0.89176 | 0.42185 | - | 1.9729 | 0.7562 |
| Pine | 0.89396 | 0.40944 | - | 2.06115 | 0.75871 |
| caffeine | 0.89802 | 0.39757 | - | 2.13358 | 0.76734 |
| soy milk | 0.89801 | 0.43179 | - | 1.99168 | 0.7705 |
| β-tocopherol | 0.89601 | 0.41418 | - | 1.89168 | 0.77255 |
| manganese | 1.11109 | 0.53181 | - | 2.66241 | 0.78716 |
| histidine | 0.90257 | 0.4148 | - | 1.90556 | 0.78747 |
| Japanese mustard | 1.11209 | 0.52371 | - | 3.27061 | 0.79437 |
| methionine | 0.90584 | 0.41495 | - | 1.90856 | 0.79508 |
| potato | 1.10645 | 0.52966 | - | 2.63818 | 0.79509 |
| Shimeji mushroom | 1.10982 | 0.52219 | - | 3.34077 | 0.79854 |
| deep-fried thin tofu | 1.09782 | 0.52446 | - | 2.69164 | 0.81156 |
| milk | 0.91891 | 0.43142 | - | 2.11445 | 0.81836 |
| Japanese mustard spinach | 0.91952 | 0.42763 | - | 2.16975 | 0.81898 |
| Niacin | 0.92311 | 0.4337 | - | 2.07064 | 0.82932 |
| Cheese | 1.08627 | 0.52077 | - | 2.56682 | 0.83074 |
| Sesame | 1.08295 | 0.51864 | - | 2.74241 | 0.8386 |
| Rice | 1.078 | 0.51403 | - | 2.4582 | 0.84489 |
| green bean | 1.07899 | 0.5111 | - | 2.83014 | 0.84694 |
| persimmon | 0.93205 | 0.43941 | - | 2.16397 | 0.84894 |
| sodium | 1.07409 | 0.50835 | - | 2.36164 | 0.85104 |
| phenylalanine | 1.07314 | 0.50358 | - | 2.28272 | 0.8517 |
| tryptophan | 1.07278 | 0.50339 | - | 2.28017 | 0.85239 |
| 22:6 n-3Docosahexaenoic acid | 1.07116 | 0.50547 | - | 2.30007 | 0.85595 |
| cooking salt | 1.06848 | 0.50983 | - | 2.43362 | 0.86277 |
| nitrate ion | 0.93832 | 0.44117 | - | 2.16458 | 0.86368 |
| retinol | 1.0677 | 0.51205 | - | 2.60845 | 0.86568 |
| Watermelon | 0.94263 | 0.45458 | - | 2.12315 | 0.87402 |
| Salt equivalent amount | 1.05779 | 0.50013 | - | 2.31833 | 0.88244 |
| vitamin B2 | 0.94607 | 0.44739 | - | 2.04114 | 0.88298 |
| threonine | 0.94661 | 0.43742 | - | 1.99689 | 0.88498 |
| peach | 1.05875 | 0.49 | - | 3.48776 | 0.88565 |
| Satsuma-age (fried fish paste) | 0.94796 | 0.45585 | - | 2.12067 | 0.88629 |
| Coffee (instant) | 0.94987 | 0.43206 | - | 2.40891 | 0.88901 |
| bread | 0.95014 | 0.44401 | - | 2.25852 | 0.89033 |
| tomato | 1.05225 | 0.50465 | - | 2.723 | 0.89539 |
| Serin | 1.04982 | 0.49104 | - | 2.22485 | 0.89753 |
| miso soup | 0.95983 | 0.45917 | - | 2.15116 | 0.9129 |
| Pickled umeboshi (pickled plums) | 1.04311 | 0.48717 | - | 3.11151 | 0.91381 |
| Total aromatic amino acids | 1.04109 | 0.48726 | - | 2.20866 | 0.9151 |
| Melon | 1.04162 | 0.49669 | - | 2.78454 | 0.916 |
| arginine | 0.96337 | 0.44292 | - | 2.02776 | 0.92161 |
| Udon | 1.02714 | 0.49169 | - | 2.32559 | 0.94386 |
| Natto | 1.02581 | 0.49289 | - | 2.4782 | 0.94676 |
| Valin | 1.02495 | 0.4795 | - | 2.17485 | 0.948 |
| zinc | 0.97655 | 0.45453 | - | 2.06317 | 0.95001 |
| safflower oil | 0.97771 | 0.45468 | - | 2.62884 | 0.95184 |
| Black tea (made from leaves) | 1.01975 | 0.47596 | - | 2.95933 | 0.9593 |
| Total sulfur-containing amino acids | 0.98112 | 0.45413 | - | 2.06876 | 0.95986 |
| Tyrosine | 1.01672 | 0.47498 | - | 2.15436 | 0.96501 |
| bean sprouts | 0.98397 | 0.47011 | - | 2.16058 | 0.96585 |
| green bell pepper | 0.98457 | 0.45811 | - | 2.63091 | 0.9669 |
| Protein by amino acid composition table | 1.01469 | 0.47328 | - | 2.14754 | 0.96922 |
| Bonito | 1.01383 | 0.48094 | - | 2.54716 | 0.97121 |
| Ramen (noodles) | 0.98718 | 0.47299 | - | 2.24253 | 0.9727 |
| isoleucine | 0.98848 | 0.46019 | - | 2.09055 | 0.97557 |
| δ-tocopherol | 0.99131 | 0.472 | - | 2.18704 | 0.98156 |
| Leucine | 0.99144 | 0.46199 | - | 2.098 | 0.98186 |
| aspartic acid | 1.00836 | 0.47031 | - | 2.13403 | 0.98243 |
| Amino acid total | 1.00656 | 0.46918 | - | 2.12979 | 0.9862 |
| water | 0.9936 | 0.47539 | - | 2.17977 | 0.98644 |
| protein | 0.99611 | 0.46393 | - | 2.10688 | 0.99178 |
| vitamin K | 0.99623 | 0.47906 | - | 2.2554 | 0.99203 |
| others | 195125.5534 | 0 | - | NA | 0.99382 |
| tea milk | 0 | NA | - | Infinite | 0.99404 |
| low fat milk | 2740048118 | 0 | - |  | 0.99412 |
| wine | 0.0469 | NA | - | 1.57287E+36 | 0.99424 |
| black tea sugar | 0.0469 | NA | - | 1.57287E+36 | 0.99424 |
| Orange juice | 1204907709 | 0 | - | NA | 0.99467 |
| soybean oil | 16.82408 | 0 | - | NA | 0.99469 |
| Apple juice | 11682.17546 | 0 | - | NA | 0.99477 |
| whiskey | 70.01269 | 0 | - | NA | 0.99511 |
| Oolong tea (made from leaves) | 0 | NA | - | Infinite | 0.99513 |
| cooking oil | NA | NA | - | NA | NA |
| 15:1pentadecenoic acid | NA | NA | - | NA | NA |
| 17:2Heptadecadienoic acid | NA | NA | - | NA | NA |
| 18:2Octadecadienoic acid | NA | NA | - | NA | NA |
| 18:3Octadecatrienoic acid | NA | NA | - | NA | NA |
| 100% grapefruit juice | NA | NA | - | NA | NA |
| corn oil | NA | NA | - | NA | NA |

NA, not available.

**Table S4.** Odds ratios for 330 food or nutrition items (adjusted for energy[per kilocalorie] and converted into z-scores) in the univariate logistic regression analyses for the risk of non-disease control, ranked according to P value.

|  | **Odds ratio** | **95% CI (low)** |  | **95% CI (high)** | **P value** |
| --- | --- | --- | --- | --- | --- |
| Chinese cabbage | 2.76201 | 1.23062 | - | 7.78953 | 0.02389 |
| Taro | 3.33803 | 1.32935 | - | 11.55501 | 0.02688 |
| meat | 0.14572 | 0.013 | - | 0.64706 | 0.04604 |
| 16:1palmitoleic acid | 0.18532 | 0.02239 | - | 0.73157 | 0.05024 |
| Wiener sausage | 0.06605 | 0.00173 | - | 0.54357 | 0.0528 |
| Shiitake mushroom | 3.28925 | 1.20876 | - | 14.20747 | 0.06102 |
| 17:1Heptadecenoic acid | 0.23692 | 0.03503 | - | 0.87206 | 0.0737 |
| 18:1n-9oleic acid | 0.16235 | 0.01412 | - | 0.83128 | 0.07408 |
| Shochu | 1.97492 | 0.93308 | - | 4.60774 | 0.08069 |
| 20:2 n-6Icosadienoic acid | 0.32843 | 0.07142 | - | 0.95891 | 0.08515 |
| Ramen (noodles) | 0.17946 | 0.01579 | - | 0.82535 | 0.08761 |
| 17:0heptadecanoic acid | 0.34588 | 0.07997 | - | 0.98093 | 0.08856 |
| 20:3 n-6Icosatrienoic acid | 0.36132 | 0.08791 | - | 0.99566 | 0.09057 |
| 16:0palmitic acid | 0.3967 | 0.11278 | - | 1.02687 | 0.09124 |
| 18:1n-7cis-vaccenic acid | 0.21608 | 0.02456 | - | 0.92777 | 0.09216 |
| Theobromine | 2.0344 | 0.93202 | - | 5.30515 | 0.09263 |
| Polyphenol | 2.0344 | 0.93202 | - | 5.30515 | 0.09263 |
| Chocolate | 2.0344 | 0.93202 | - | 5.30515 | 0.09263 |
| monounsaturated fatty acids | 0.35646 | 0.08307 | - | 0.99933 | 0.09359 |
| 18:1Total | 0.36251 | 0.08491 | - | 1.01431 | 0.09863 |
| Molybdenum | 1.98181 | 0.90826 | - | 5.06593 | 0.10038 |
| Total amount of fatty acids | 0.44267 | 0.13647 | - | 1.10415 | 0.1155 |
| 20:1Icosenoic acid | 0.45558 | 0.1462 | - | 1.11156 | 0.11611 |
| Lipid | 0.44981 | 0.14229 | - | 1.11056 | 0.11636 |
| Triacylglycerol equivalent | 0.44454 | 0.13754 | - | 1.10726 | 0.11656 |
| 22:4 n-6docosatetraenoic acid | 0.36983 | 0.08606 | - | 1.07093 | 0.11962 |
| 18:0stearic acid | 0.45057 | 0.13467 | - | 1.1401 | 0.13326 |
| Sesame | 1.83236 | 0.85573 | - | 4.69879 | 0.13467 |
| saturated fatty acids | 0.46522 | 0.14408 | - | 1.16193 | 0.14078 |
| Alcohol | 1.78727 | 0.82 | - | 4.0722 | 0.143 |
| Japanese tea (cans/plastic bottles) | 0.02976 | 0.00004 | - | 0.84155 | 0.14496 |
| Stir-fried beef | 0.10739 | 0.0021 | - | 0.95586 | 0.14753 |
| Potassium | 1.79008 | 0.8147 | - | 4.21219 | 0.15005 |
| Magnesium | 1.81021 | 0.8167 | - | 4.37977 | 0.1511 |
| Ash | 1.8093 | 0.81103 | - | 4.37796 | 0.1548 |
| grilled chicken | 0.27805 | 0.03035 | - | 1.06141 | 0.15844 |
| Yogurt | 1.74176 | 0.81724 | - | 4.32343 | 0.15941 |
| 14:1myristoleic acid | 0.42789 | 0.10078 | - | 1.17203 | 0.16616 |
| α-carotene | 1.73258 | 0.80124 | - | 4.21232 | 0.16699 |
| Luxury drinks | 0.44672 | 0.10085 | - | 1.19158 | 0.17928 |
| Wasabi | 1.72047 | 0.78049 | - | 4.03773 | 0.1797 |
| Biotin | 1.74037 | 0.78332 | - | 4.24152 | 0.18098 |
| Natto | 1.66493 | 0.77787 | - | 3.91067 | 0.1854 |
| Daidzein | 1.70466 | 0.77846 | - | 4.11253 | 0.18593 |
| Copper | 1.72417 | 0.78047 | - | 4.25446 | 0.18596 |
| Oils and fats | 0.28593 | 0.02724 | - | 1.14463 | 0.18783 |
| Genistein | 1.69965 | 0.7743 | - | 4.09956 | 0.18965 |
| Cabbage | 1.68393 | 0.77167 | - | 3.94477 | 0.19013 |
| Peach | 0.02037 | 0 | - | 0.84093 | 0.19158 |
| grilled beef | 0.01303 | 0 | - | 0.62186 | 0.19208 |
| 20:4 n-6arachidonic acid | 0.57914 | 0.23233 | - | 1.31291 | 0.20276 |
| vitamin K | 1.63133 | 0.74964 | - | 3.66519 | 0.20774 |
| Bacon | 0.36578 | 0.04147 | - | 1.16637 | 0.20845 |
| tap water | 1.62417 | 0.74486 | - | 3.60671 | 0.21174 |
| Bonito | 0.21497 | 0.01131 | - | 1.12151 | 0.21216 |
| Banana | 1.75005 | 0.80284 | - | 5.8397 | 0.21409 |
| daikon (Japanese white radish) | 1.64805 | 0.74235 | - | 3.84657 | 0.21868 |
| fried chicken | 0.4339 | 0.07571 | - | 1.24234 | 0.22223 |
| Whitebait | 0.37427 | 0.05221 | - | 1.23625 | 0.2227 |
| organic acid | 1.62843 | 0.75363 | - | 4.19547 | 0.22407 |
| Lettuce | 1.617 | 0.73655 | - | 3.70375 | 0.22412 |
| Mushrooms | 1.82053 | 0.81862 | - | 7.48612 | 0.22512 |
| stir-fried chicken | 0.50402 | 0.12105 | - | 1.2941 | 0.22965 |
| 20:0arachidic acid | 0.56514 | 0.19677 | - | 1.34126 | 0.23262 |
| rice cake | 0.2065 | 0.00336 | - | 1.03089 | 0.233 |
| water soluble dietary fiber | 1.60333 | 0.72889 | - | 3.80884 | 0.23669 |
| Stir-fried pork | 0.42209 | 0.07206 | - | 1.28195 | 0.23788 |
| Histidine | 0.60158 | 0.24265 | - | 1.36885 | 0.23791 |
| Pickled umeboshi (pickled plums) | 1.67696 | 0.77328 | - | 5.79425 | 0.23926 |
| Glycine | 0.60802 | 0.24498 | - | 1.38353 | 0.24778 |
| Carrot | 1.5816 | 0.72025 | - | 3.73597 | 0.24831 |
| Sodium | 1.62442 | 0.71919 | - | 4.00874 | 0.25176 |
| Garlic | 2.1173 | 0.87955 | - | 14.68531 | 0.25235 |
| Kamaboko (boiled fish paste) | 0.17464 | 0.00266 | - | 1.13779 | 0.25657 |
| Pickled takuwan (pickled daikon radish) | 0.01479 | 0 | - | 1.10975 | 0.25754 |
| Salt equivalent amount | 1.61102 | 0.71303 | - | 3.97222 | 0.25983 |
| Stewed pork | 0.38006 | 0.04042 | - | 1.25195 | 0.26067 |
| Retinol | 0.41444 | 0.05374 | - | 1.27278 | 0.26193 |
| green asparagus | 1.56461 | 0.72032 | - | 4.13427 | 0.26346 |
| Total amount of dietary fiber | 1.56198 | 0.70688 | - | 3.67095 | 0.26353 |
| Enoki mushroom | 1.56592 | 0.72676 | - | 4.55521 | 0.26398 |
| Watermelon | 0.15073 | 0.00072 | - | 0.9804 | 0.26444 |
| Canned tuna | 0.48828 | 0.09947 | - | 1.32526 | 0.26466 |
| Persimmon | 0.27757 | 0.01012 | - | 1.2046 | 0.26635 |
| salad dressing | 1.56349 | 0.7036 | - | 3.59794 | 0.26649 |
| insoluble dietary fiber | 1.5519 | 0.70071 | - | 3.6158 | 0.27127 |
| Vegetables | 1.51797 | 0.69774 | - | 3.44147 | 0.27237 |
| Shimeji mushroom | 1.54939 | 0.71353 | - | 4.3867 | 0.27832 |
| vegetable juice | 1.54329 | 0.71098 | - | 4.44538 | 0.28229 |
| Coffee (instant) | 1.54136 | 0.71068 | - | 4.54904 | 0.28482 |
| Pickled turnips | 1.4991 | 0.68733 | - | 3.31516 | 0.28496 |
| Japanese mustard | 1.53337 | 0.7047 | - | 4.23593 | 0.28571 |
| Shrimp | 1.51295 | 0.69329 | - | 3.66201 | 0.28602 |
| Milk | 0.31879 | 0.01405 | - | 1.24985 | 0.28632 |
| sweet potato | 1.51058 | 0.69086 | - | 3.54334 | 0.28666 |
| strawberry | 1.83713 | 0.80723 | - | 18.7326 | 0.2887 |
| Chikuwa (tubular fish-paste) | 0.45395 | 0.06097 | - | 1.33447 | 0.29435 |
| vitamin B12 | 1.54344 | 0.70831 | - | 5.46687 | 0.29902 |
| green and yellow vegetables | 1.48708 | 0.68064 | - | 3.53091 | 0.30144 |
| lycopene | 1.49566 | 0.68555 | - | 3.85381 | 0.30223 |
| calcium | 1.50392 | 0.67626 | - | 3.46363 | 0.30632 |
| Boiled pork | 0.4769 | 0.067 | - | 1.37902 | 0.31522 |
| Cod roe, sujiko(salted salmon roe), salmon roe | 0.45088 | 0.04784 | - | 1.36394 | 0.31917 |
| Japanese tea (made from leaves) | 0.54627 | 0.09807 | - | 1.41058 | 0.32182 |
| Rice crackers | 1.4682 | 0.67146 | - | 4.09283 | 0.32499 |
| Coffee (cans/plastic bottles) | 0.13702 | 0.00012 | - | 1.04532 | 0.32604 |
| Sauce | 0.56187 | 0.12268 | - | 1.42972 | 0.32672 |
| miso soup | 1.46153 | 0.65998 | - | 3.27756 | 0.3285 |
| pork liver | 0.46382 | 0.04558 | - | 1.36929 | 0.33078 |
| Kiwi fruit | 1.43267 | 0.65576 | - | 3.48216 | 0.33119 |
| Seasonings/Spices | 1.48535 | 0.65961 | - | 3.49762 | 0.33758 |
| δ-tocopherol | 1.46517 | 0.65517 | - | 3.36129 | 0.34086 |
| Retinol activity equivalent | 0.57603 | 0.13573 | - | 1.45592 | 0.34206 |
| bread | 0.46708 | 0.05962 | - | 1.41098 | 0.34544 |
| 24:0lignoceric acid | 0.6094 | 0.17369 | - | 1.47878 | 0.34651 |
| β-cryptoxanthin | 0.36353 | 0.02119 | - | 1.35746 | 0.34784 |
| 14:0myristic acid | 0.60498 | 0.17735 | - | 1.48527 | 0.34947 |
| Yam/Chinese yam | 0.58284 | 0.13197 | - | 1.47283 | 0.35799 |
| beef steak | 0.38539 | 0.01099 | - | 1.34298 | 0.35869 |
| Japanese pear | 0.22985 | 0.00367 | - | 1.30623 | 0.35937 |
| Butter | 0.00028 | 0 | - | 0.37128 | 0.36087 |
| carbonated drinks | 0.59172 | 0.12048 | - | 1.47684 | 0.36127 |
| caffeine | 1.41485 | 0.63931 | - | 3.70321 | 0.36258 |
| Milks | 1.43349 | 0.64132 | - | 3.23032 | 0.36284 |
| nori seaweed | 1.41393 | 0.63576 | - | 3.14872 | 0.36653 |
| tannin | 1.40782 | 0.63474 | - | 3.6668 | 0.36876 |
| apple | 0.63619 | 0.18832 | - | 1.51512 | 0.37018 |
| Octopus | 1.39758 | 0.6282 | - | 3.22807 | 0.37679 |
| fried pork | 0.58665 | 0.12571 | - | 1.49408 | 0.37704 |
| Tara | 1.40688 | 0.62985 | - | 3.13846 | 0.37985 |
| manganese | 0.62445 | 0.1632 | - | 1.51578 | 0.38086 |
| 15:0pentadecanoic acid | 0.63478 | 0.19389 | - | 1.52836 | 0.38116 |
| grapes | 2.52507 | 0.89741 | - | 65.05349 | 0.38154 |
| Snacks (potato chips) | 0.46442 | 0.01993 | - | 1.40738 | 0.38614 |
| pickled green leaves | 0.56549 | 0.07978 | - | 1.48379 | 0.39011 |
| beans | 1.40857 | 0.62646 | - | 3.16194 | 0.39353 |
| egg | 1.42049 | 0.62876 | - | 3.3174 | 0.3955 |
| eggs | 1.42049 | 0.62876 | - | 3.3174 | 0.3955 |
| pantothenic acid | 1.40543 | 0.62393 | - | 3.2342 | 0.4003 |
| Japanese mustard spinach | 1.36435 | 0.60899 | - | 3.13561 | 0.40238 |
| Pickled cucumber | 1.36181 | 0.60776 | - | 3.08124 | 0.40369 |
| 7:0heptanoic acid | 1.37898 | 0.61483 | - | 3.04232 | 0.40624 |
| 13:0tridecanoic acid | 1.37839 | 0.61449 | - | 3.04101 | 0.40691 |
| 12:0Lauric acid | 0.62211 | 0.14788 | - | 1.54829 | 0.41714 |
| iron | 1.3988 | 0.61816 | - | 3.29796 | 0.41748 |
| mandarin orange | 0.46826 | 0.0304 | - | 1.46539 | 0.41958 |
| noodle soup | 0.59908 | 0.11012 | - | 1.53574 | 0.421 |
| 22:0Behenic acid | 0.67771 | 0.21791 | - | 1.58625 | 0.42525 |
| alanine | 0.7195 | 0.30495 | - | 1.63023 | 0.42985 |
| Broccoli | 1.3416 | 0.59318 | - | 3.0641 | 0.43071 |
| Ketchup | 0.59531 | 0.09019 | - | 1.53664 | 0.43142 |
| Other vegetables | 1.35475 | 0.60037 | - | 3.13429 | 0.43267 |
| Confectionery | 1.34267 | 0.59332 | - | 3.1162 | 0.43782 |
| Margarine | 0.50781 | 0.03609 | - | 1.50868 | 0.43997 |
| soba (buckwheat noodles) | 0.6812 | 0.20106 | - | 1.599 | 0.44203 |
| β-carotene | 1.33667 | 0.59114 | - | 2.89704 | 0.4466 |
| Mayonnaise | 0.67655 | 0.18926 | - | 1.59907 | 0.44693 |
| bean sprouts | 1.3481 | 0.59615 | - | 3.01351 | 0.45488 |
| bird liver | 0.50821 | 0.03122 | - | 1.52533 | 0.45592 |
| Sugar/sweeteners | 0.62646 | 0.08994 | - | 1.57203 | 0.45646 |
| methionine | 0.73418 | 0.31352 | - | 1.66395 | 0.4565 |
| n-6 polyunsaturated fatty acids | 0.71884 | 0.2737 | - | 1.64483 | 0.45879 |
| 22:5 n-3Docosapentaenoic acid | 0.73434 | 0.30144 | - | 1.66809 | 0.46707 |
| chromium | 1.33515 | 0.58894 | - | 3.04493 | 0.47072 |
| Pine | 0.60311 | 0.0644 | - | 1.58076 | 0.47769 |
| polyunsaturated fatty acids | 0.73299 | 0.28602 | - | 1.67202 | 0.47951 |
| polyunsaturated fatty acids | 0.73338 | 0.28622 | - | 1.67282 | 0.4802 |
| Green onion/Wakegi (tree onion) | 0.36063 | 0.00928 | - | 1.51802 | 0.48286 |
| 18:2n-6linoleic acid | 0.73454 | 0.28135 | - | 1.67667 | 0.48649 |
| 24:1Tetracosenoic acid | 0.74784 | 0.31393 | - | 1.69731 | 0.48863 |
| coffee sugar | 0.65428 | 0.09333 | - | 1.61219 | 0.49121 |
| Beef stew | 0.67099 | 0.14102 | - | 1.62646 | 0.49123 |
| Commercial water | 0.68499 | 0.14719 | - | 1.63326 | 0.49217 |
| arginine | 0.75603 | 0.3285 | - | 1.7157 | 0.49566 |
| tomato | 1.28835 | 0.5585 | - | 2.78803 | 0.49758 |
| Pickles | 1.29826 | 0.56863 | - | 2.81398 | 0.50061 |
| Dried fish | 1.29698 | 0.56846 | - | 2.81618 | 0.50561 |
| pork soup | 0.70721 | 0.17311 | - | 1.66018 | 0.51141 |
| vitamin D | 0.75652 | 0.31127 | - | 1.71882 | 0.51146 |
| Yakult | 1.27544 | 0.55016 | - | 2.77548 | 0.518 |
| cooking salt | 1.28967 | 0.56502 | - | 2.87913 | 0.52219 |
| Japanese sake | 1.2632 | 0.5375 | - | 2.72745 | 0.52684 |
| jam | 0.10506 | 0 | - | 1.72235 | 0.52684 |
| Potatoes | 1.27563 | 0.55292 | - | 2.8673 | 0.52711 |
| pacific saury | 0.75088 | 0.26305 | - | 1.71198 | 0.53097 |
| 18:4 n-3Octadecatetraenoic acid | 0.76319 | 0.29783 | - | 1.73395 | 0.53598 |
| protein | 0.77665 | 0.33261 | - | 1.76513 | 0.54206 |
| Rice | 1.27365 | 0.55648 | - | 2.84829 | 0.54375 |
| Black tea (made from leaves) | 0.17588 | 0.00002 | - | 1.57034 | 0.54611 |
| Cheese | 1.25871 | 0.54563 | - | 2.73496 | 0.5553 |
| proline | 0.77064 | 0.29437 | - | 1.75194 | 0.55624 |
| Vitamin B1 | 0.77211 | 0.29865 | - | 1.75382 | 0.55645 |
| Amino acid total | 0.78399 | 0.33672 | - | 1.78292 | 0.55687 |
| 22:5 n-6Docosapentaenoic acid | 0.77901 | 0.31616 | - | 1.76751 | 0.55896 |
| 22:1docosenoic acid | 0.77266 | 0.29257 | - | 1.75314 | 0.55941 |
| Udon | 0.76625 | 0.26232 | - | 1.7423 | 0.56417 |
| glutamic acid | 0.78185 | 0.31745 | - | 1.77504 | 0.56486 |
| lysine | 0.79121 | 0.33984 | - | 1.80035 | 0.57213 |
| Protein by amino acid composition table | 0.79165 | 0.34074 | - | 1.8016 | 0.5724 |
| threonine | 0.79285 | 0.34185 | - | 1.80472 | 0.5747 |
| Total sulfur-containing amino acids | 0.79665 | 0.34618 | - | 1.81543 | 0.5808 |
| Beer | 0.71261 | 0.08814 | - | 1.70564 | 0.581 |
| selenium | 0.79393 | 0.33491 | - | 1.80535 | 0.58103 |
| β-carotene equivalent | 1.23618 | 0.52804 | - | 2.66518 | 0.5813 |
| soy milk | 1.23339 | 0.52682 | - | 2.6176 | 0.5857 |
| phosphorus | 1.25144 | 0.54864 | - | 2.90469 | 0.58609 |
| green bell pepper | 0.70432 | 0.05692 | - | 1.71325 | 0.59935 |
| Clams | 3.06725 | 0.85149 | - | NA | 0.59961 |
| n-3 polyunsaturated fatty acids | 0.80223 | 0.33419 | - | 1.82218 | 0.60139 |
| pumpkin | 1.22106 | 0.51973 | - | 2.64189 | 0.60601 |
| olive oil | 1.22007 | 0.52025 | - | 2.61721 | 0.60863 |
| fruits | 1.21169 | 0.50437 | - | 2.6609 | 0.61255 |
| 22:2docosadienoic acid | 1.21407 | 0.52595 | - | 2.68012 | 0.62952 |
| Salmon | 1.21407 | 0.52595 | - | 2.68012 | 0.62952 |
| coffee milk | 0.28967 | NA | - | 1.78937 | 0.6365 |
| squid | 1.20152 | 0.51036 | - | 2.58024 | 0.63922 |
| 20:4 n-3Icosatetraenoic acid | 0.82842 | 0.34976 | - | 1.88324 | 0.65289 |
| water | 1.18838 | 0.51272 |  | 2.62959 | 0.6689 |
| onion | 1.1758 | 0.47749 | - | 2.54929 | 0.6713 |
| 10:0Decanoic acid | 0.82011 | 0.26216 | - | 1.84213 | 0.67263 |
| boiled chicken | 0.82593 | 0.27847 | - | 1.84647 | 0.67372 |
| 20:5 n-3Icosapentaenoic acid | 0.83894 | 0.35269 | - | 1.90508 | 0.67547 |
| 21:5 n-3henicosapentaenoic acid | 0.83701 | 0.33596 | - | 1.88827 | 0.67733 |
| tofu | 0.76836 | 0.06889 | - | 1.80556 | 0.67806 |
| Wakame seaweed | 0.82448 | 0.25309 | - | 1.83964 | 0.67862 |
| Leucine | 0.84495 | 0.3655 | - | 1.93103 | 0.68391 |
| folic acid | 1.17785 | 0.50538 | - | 2.63401 | 0.68423 |
| sea ​​bream | 1.17414 | 0.49824 | - | 2.55935 | 0.68636 |
| 18:3 n-3α-linolenic acid | 0.84057 | 0.3321 | - | 1.894 | 0.68685 |
| grains | 0.84267 | 0.33536 | - | 1.8993 | 0.68978 |
| tryptophan | 0.84982 | 0.36874 | - | 1.94455 | 0.69374 |
| Serin | 0.85085 | 0.37056 | - | 1.94923 | 0.6954 |
| 22:6 n-3Docosahexaenoic acid | 0.85258 | 0.36127 | - | 1.9391 | 0.70268 |
| nitrate ion | 1.15688 | 0.47107 | - | 2.52071 | 0.70694 |
| iodine | 1.15964 | 0.48443 | - | 2.55254 | 0.70725 |
| phenylalanine | 0.85739 | 0.37203 | - | 1.96318 | 0.70971 |
| 8:0octanoic acid | 0.84264 | 0.27231 | - | 1.87942 | 0.71077 |
| cholesterol | 0.8617 | 0.37932 | - | 1.98602 | 0.7168 |
| 16:0isopalmitic acid | 0.84628 | 0.27432 | - | 1.88566 | 0.71697 |
| Bisket | 1.15265 | 0.48232 | - | 2.49284 | 0.72027 |
| deep-fried thin tofu | 0.85061 | 0.27996 | - | 1.88958 | 0.72204 |
| isoleucine | 0.86606 | 0.37627 | - | 1.98448 | 0.72793 |
| Japanese sweets | 0.85595 | 0.28032 | - | 1.89649 | 0.73142 |
| vitamin C | 1.13637 | 0.45898 | - | 2.47425 | 0.74365 |
| Shungiku (garland chrysanthemum) | 0.8333 | 0.07625 | - | 1.88417 | 0.74395 |
| green bean | 0.85927 | 0.25007 | - | 1.90632 | 0.7471 |
| Total aromatic amino acids | 0.87893 | 0.38255 | - | 2.01735 | 0.75482 |
| Satsuma-age (fried fish paste) | 0.87128 | 0.30168 | - | 1.92311 | 0.75527 |
| Japanese leek | 1.12359 | 0.41949 | - | 2.44181 | 0.7603 |
| 16:2Hexadecadienoic acid | 0.88025 | 0.36534 | - | 1.98959 | 0.76178 |
| Peanuts | 0.87538 | 0.28309 | - | 1.92384 | 0.76662 |
| vitamin B6 | 1.12813 | 0.48138 | - | 2.5048 | 0.76699 |
| seafood | 1.12916 | 0.48902 | - | 2.58018 | 0.7678 |
| Available carbohydrates (monosaccharide equivalents) | 1.12642 | 0.48211 | - | 2.54642 | 0.77035 |
| carbohydrates | 1.12705 | 0.4875 | - | 2.57754 | 0.77124 |
| Chive | 0.86161 | 0.12349 | - | 1.91955 | 0.77337 |
| 10:1Decenoic acid | 0.88248 | 0.30535 | - | 1.95167 | 0.77841 |
| 4:0butyric acid | 0.88379 | 0.29595 | - | 1.9513 | 0.78255 |
| Tyrosine | 0.8923 | 0.38857 | - | 2.05019 | 0.78284 |
| potato | 1.11503 | 0.45951 | - | 2.41333 | 0.78556 |
| 16:4Hexadecatetraenoic acid | 0.89431 | 0.37002 | - | 2.01696 | 0.79055 |
| Eggplant | 0.87592 | 0.13141 | - | 1.94108 | 0.79236 |
| soup | 1.10976 | 0.45622 |  | 2.40412 | 0.79492 |
| α-tocopherol | 1.11136 | 0.47416 | - | 2.48694 | 0.79596 |
| Burdock | 0.89699 | 0.34329 | - | 1.98729 | 0.79972 |
| coffee (made from beans) | 1.10346 | 0.42752 | - | 2.37041 | 0.80206 |
| algae | 0.89664 | 0.31276 | - | 1.96759 | 0.80318 |
| 6:0hexanoic acid | 0.89633 | 0.3107 | - | 1.97697 | 0.80419 |
| 15:0 antpentadecanoic acid | 0.90059 | 0.31612 | - | 1.98442 | 0.81145 |
| Seeds and fruits | 0.90303 | 0.30076 | - | 1.97158 | 0.81669 |
| Valin | 0.91301 | 0.39777 | - | 2.10119 | 0.82591 |
| Konjac | 1.0885 | 0.42405 | - | 2.38292 | 0.83058 |
| spinach | 1.08732 | 0.44464 | - | 2.36043 | 0.83571 |
| Mixed oil | 0.91543 | 0.30218 | - | 2.00218 | 0.84095 |
| 17:0 antheptadecanoic acid | 0.91849 | 0.33002 | - | 2.0197 | 0.84443 |
| zinc | 0.92269 | 0.40341 | - | 2.12752 | 0.84579 |
| Low ham | 0.92186 | 0.3163 | - | 2.00894 | 0.8514 |
| acetic acid | 1.07947 | 0.4541 | - | 2.38612 | 0.85163 |
| γ-tocopherol | 1.07937 | 0.46116 | - | 2.43658 | 0.85262 |
| Cystine | 0.92651 | 0.40429 | - | 2.1397 | 0.85356 |
| spaghetti macaroni | 1.07501 | 0.42766 | - | 2.33836 | 0.85729 |
| cucumber | 0.92532 | 0.24409 | - | 2.01757 | 0.8631 |
| Salt cod/salt salmon | 1.07179 | 0.4251 | - | 2.30404 | 0.86344 |
| Tomato juice | 1.06545 | 0.38221 | - | 2.29045 | 0.87296 |
| β-tocopherol | 0.93678 | 0.40704 | - | 2.15432 | 0.87478 |
| deep-fried thick tofu | 0.94216 | 0.32754 | - | 2.0579 | 0.88991 |
| 18:3n-6γ-linolenic acid | 0.94327 | 0.36428 | - | 2.07819 | 0.89028 |
| alcoholic drinks | 1.05282 | 0.38699 | - | 2.27072 | 0.89797 |
| vitamin B2 | 0.94992 | 0.39254 | - | 2.12997 | 0.90222 |
| ice cream | 1.04825 | 0.39288 | - | 2.2346 | 0.90722 |
| Hijiki seaweed | 0.95252 | 0.33178 | - | 2.07484 | 0.90942 |
| aspartic acid | 0.96002 | 0.42146 | - | 2.22836 | 0.92149 |
| citrus | 0.96208 | 0.28493 | - | 2.06666 | 0.92819 |
| Cake | 1.03775 | 0.43123 | - | 2.29487 | 0.92827 |
| boiled tofu | 0.96573 | 0.2932 | - | 2.10244 | 0.93516 |
| Horse mackerel | 0.96856 | 0.38762 | - | 2.13839 | 0.93913 |
| Somen/Hiyamugi | 0.96868 | 0.37485 | - | 2.11599 | 0.93952 |
| pickled eggplant | 0.97252 | 0.3442 | - | 2.09621 | 0.94727 |
| Fruit drinks (not 100%) | 0.97439 | 0.25137 | - | 2.10321 | 0.95153 |
| ammonia | 0.97587 | 0.41036 | - | 2.20678 | 0.95319 |
| 16:3Hexadecatrienoic acid | 0.97709 | 0.40788 | - | 2.19253 | 0.95558 |
| Pickled Chinese cabbage | 1.02036 | 0.40995 | - | 2.22275 | 0.96101 |
| moisture | 0.98596 | 0.42209 | - | 2.24883 | 0.97282 |
| Niacin | 0.98603 | 0.36982 | - | 2.15263 | 0.97313 |
| Yellowtail | 1.0044 | 0.3234 | - | 2.17837 | 0.99153 |
| gross weight | 1.00385 | 0.43175 | - | 2.29674 | 0.99261 |
| Oolong tea (cans/plastic bottles) | 0 | NA | - | 5.10622E+76 | 0.99403 |
| others | 0.00001 | NA | - | 1.8643E+103 | 0.99407 |
| safflower oil | 0.00461 | NA | - | 1.14799E+49 | 0.99432 |
| tea milk | 0 | NA | - | Infinite | 0.99438 |
| low fat milk | 0 | NA | - | 3.7495E+161 | 0.99442 |
| Eel | 0 | NA | - | 9.9678E+151 | 0.99452 |
| Rapeseed oil/canola oil | 0 | NA | - | 6.793E+172 | 0.99459 |
| Black tea (cans/plastic bottles) | 0 | NA | - | 1.92E+97 | 0.99465 |
| Orange juice | 0 | NA | - | 2.1367E+204 | 0.99483 |
| wine | 0.06445 | NA | - | 2.13569E+36 | 0.99484 |
| Koya tofu (freeze-dried tofu) | 0 | NA | - | 1.1261E+152 | 0.99484 |
| black tea sugar | 0.06445 | NA | - | 2.13569E+36 | 0.99484 |
| soybean oil | 0.06445 | NA | - | 2.13569E+36 | 0.99484 |
| Apple juice | 0.00011 | NA | - | 5.17592E+94 | 0.99499 |
| whiskey | 0.01607 | NA | - | 2.21584E+45 | 0.99524 |
| Melon | 0 | NA | - | Infinite | 0.99572 |
| Oolong tea (made from leaves) | 0 | NA | - | Infinite | 0.99583 |
| cooking oil | NA | NA | - | NA | NA |
| 15:1pentadecenoic acid | NA | NA | - | NA | NA |
| 17:2Heptadecadienoic acid | NA | NA | - | NA | NA |
| 18:2Octadecadienoic acid | NA | NA | - | NA | NA |
| 18:3Octadecatrienoic acid | NA | NA | - | NA | NA |
| 100% grapefruit juice | NA | NA | - | NA | NA |
| corn oil | NA | NA | - | NA | NA |

NA, not available.
