# Supplementary figures and images for: Vinegar intake in patients undergoing immune checkpoint inhibitor therapy: food frequency questionnaire study
Source: Front Immunol. 2025 Dec 11;16:1640603. doi: 10.3389/fimmu.2025.1640603 (PMC12738812; doi:10.3389/fimmu.2025.1640603)

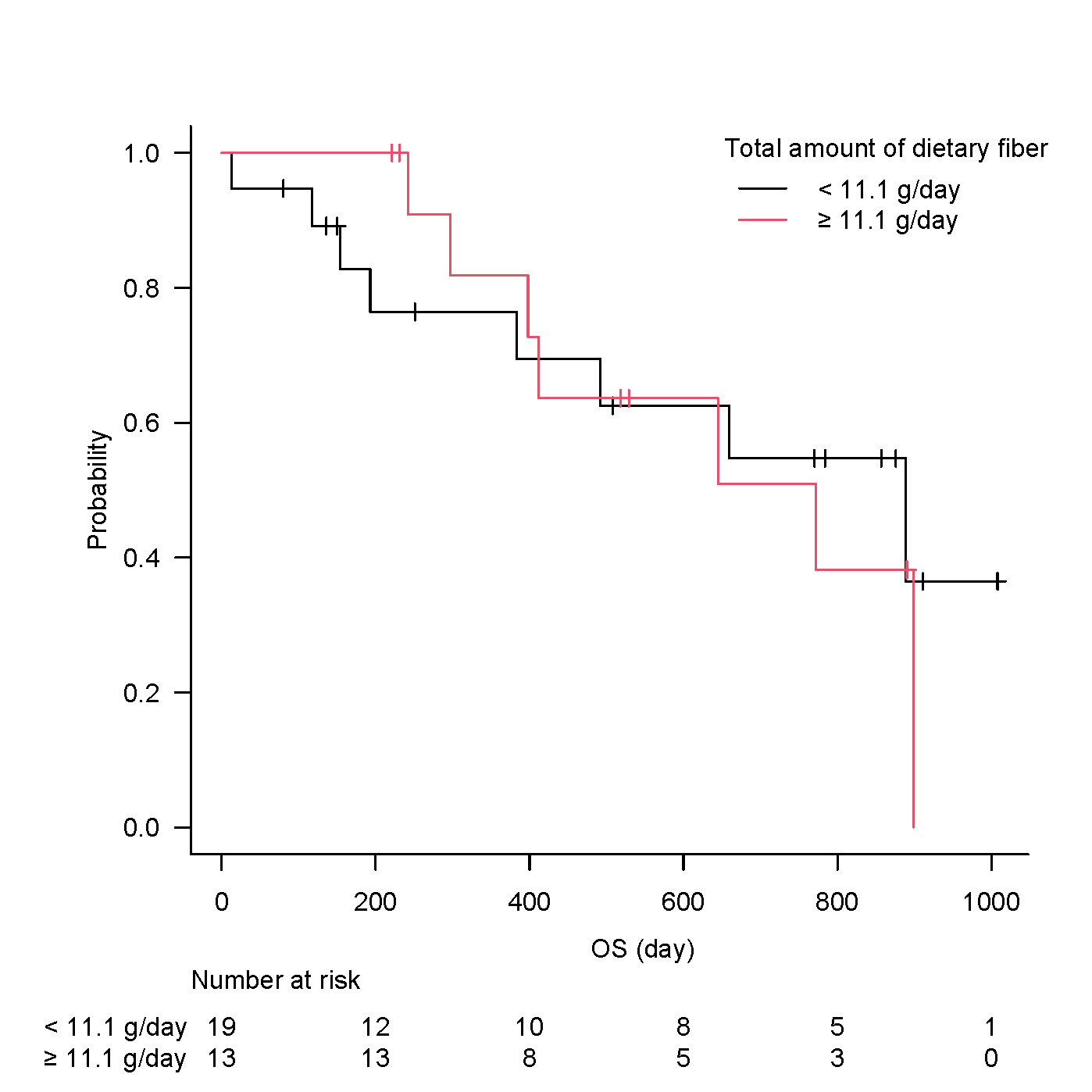

Supplement: Supplementary file 2 [file Image1.tiff]

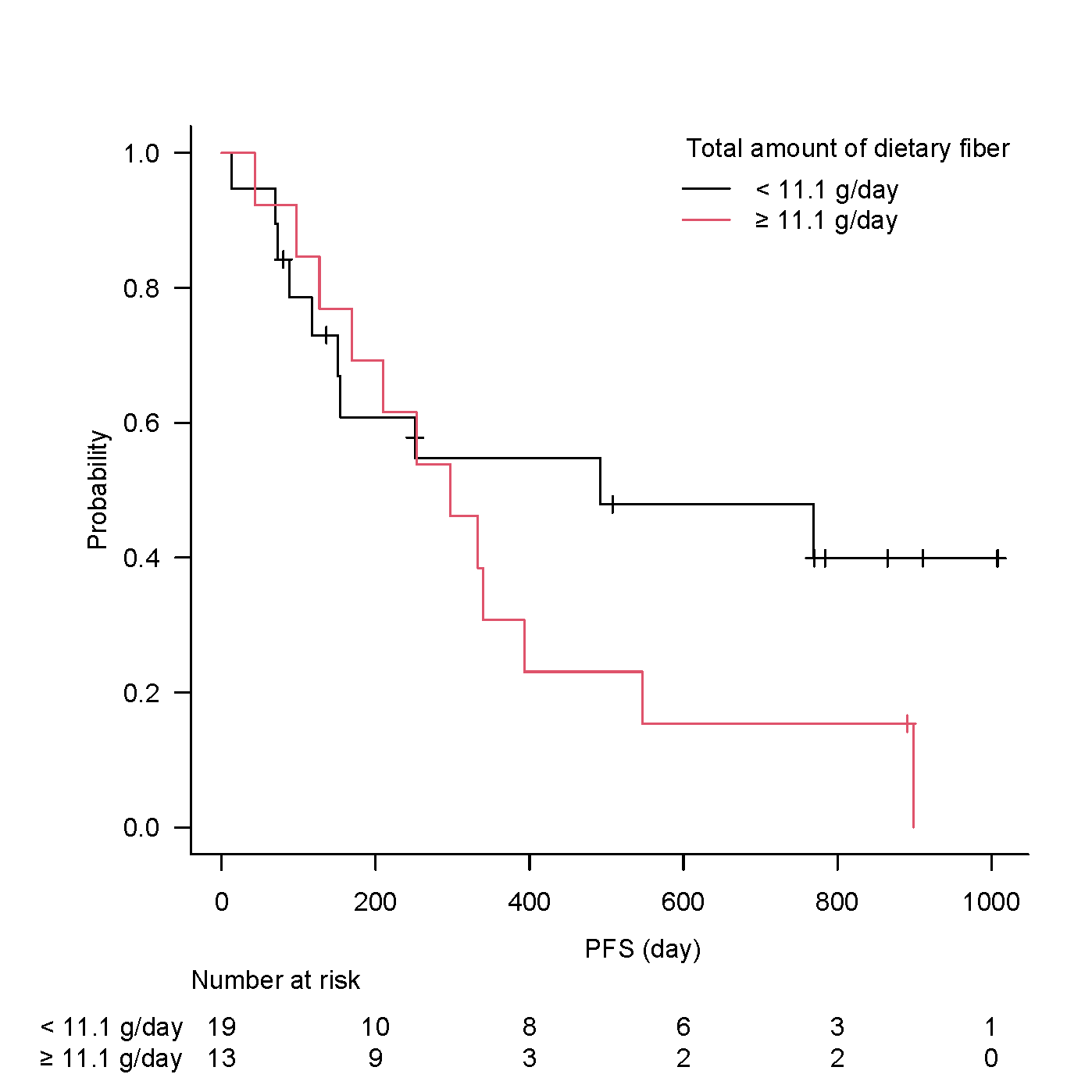

Supplement: Supplementary file 3 [file Image2.tiff]
